# The association between DXA‐derived body fat measures and breast cancer risk among postmenopausal women in the Women's Health Initiative

**DOI:** 10.1002/cam4.2690

**Published:** 2019-12-25

**Authors:** Rhonda S. Arthur, Xiaonan Xue, Victor Kamensky, Rowan T. Chlebowski, Michael Simon, Juhua Luo, Aladdin H. Shadyab, Marian L. Neuhouser, Hailey Banack, Gloria Y. F. Ho, Dorothy S. Lane, Kathy Pan, Kerryn W. Reding, Sylvia Wassertheil‐Smoller, Andrew J. Dannenberg, Thomas E. Rohan

**Affiliations:** ^1^ Department of Epidemiology and Population Health Albert Einstein College of Medicine Bronx NY USA; ^2^ Los Angeles Biomedical Research Institute at Harbor‐UCLA Medical Center Torrance CA USA; ^3^ Department of Oncology Karmanos Cancer Institute Detroit MI USA; ^4^ Departments of Epidemiology and Biostatistics School of Public Health Indiana University Bloomington Bloomington IN USA; ^5^ Division of Epidemiology Department of Family Medicine and Public Health University of California San Diego School of Medicine La Jolla CA USA; ^6^ Cancer Prevention Program Fred Hutchinson Cancer Research Center Seattle WA USA; ^7^ Department of Epidemiology and Environmental Health School of Public Health and Health Professions University at Buffalo The State University of New York Buffalo NY USA; ^8^ Feinstein Institute for Medical Research Northwell Health, and Donald and Barbara Zucker School of Medicine at Hofstra/Northwell Great Neck NY USA; ^9^ Department of Preventive Medicine Stony Brook University School of Medicine Stony Brook NY USA; ^10^ University of Washington School of Nursing Seattle WA USA; ^11^ Weill Cornell Medical College New York NY USA

**Keywords:** body fat, breast cancer risk, postmenopausal women

## Abstract

**Background:**

Most studies demonstrating an association between excess adiposity and postmenopausal breast cancer have used anthropometric measures, particularly body mass index (BMI). However, more direct body fat measures may more accurately determine the relationship between body fat distribution and breast cancer risk.

**Methods:**

Cox proportional hazards regression models were created to examine the associations of dual‐energy x‐ray absorptiometry (DXA) body fat measures (at baseline and during follow‐up) with breast cancer risk among 10 931 postmenopausal women from the Women's Health Initiative cohort. A total of 639 incident invasive breast cancer cases (including 484 estrogen receptor positive (ER+) cases) were ascertained after a median follow‐up of 15.0 years.

**Results:**

Excess whole body fat mass and trunk fat mass were positively associated with risk invasive breast cancer risk. These associations persisted even after additional adjustment for standard anthropometric measures. In time‐dependent analyses, we observed that both whole body fat mass and trunk fat mass, in the highest versus lowest category, were associated with a doubling of risk of invasive breast cancer overall (HR: 2.17; 95% CI: 1.54‐3.05 and 2.20; 1.55‐3.14, respectively) and of ER+ breast cancer (2.05; 1.37‐3.05 and 2.03; 1.34‐3.07, respectively). The remaining DXA measures were also positively associated with breast cancer risk in baseline and time‐dependent analyses.

**Conclusion:**

These findings suggest that DXA‐derived body fat measures are positively associated with breast cancer risk after adjustment for BMI and other conventional breast cancer risk factors.

## Graphical abstract

Our study demonstrated that DXA‐derived body fat measures were positively associated with breast cancer risk after adjustment for BMI and other conventional breast cancer risk factors. These findings suggest that DXA‐derived measures of overall and central adiposity may explain invasive breast cancer risk beyond that of BMI and other risk factors.

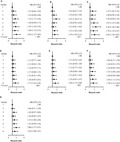

## 1 INTRODUCTION

Excess adiposity is believed to contribute to carcinogenesis through several mechanisms, including chronic inflammation, hyperinsulinemia, elevated leptin and reduced adiponectin levels, hyperlipidemia and abnormal sex steroid hormone metabolism.^1^, ^2^ The preponderance of epidemiologic studies examining the association of body fat with risk of breast cancer (including hormone receptor‐positive breast cancer) have used body mass index (BMI) as the exposure of interest.^3^, ^4^, ^5^, ^6^, ^7^, ^8^ These studies have provided consistent evidence to support a role for excess adiposity, as defined by having a BMI >25.0 kg/m^2^ (overweight/obesity), in the development of breast cancer among postmenopausal women.^3^, ^4^, ^5^, ^6^, ^7^, ^8^

As a measure of adiposity, BMI is limited, in that it does not truly reflect one's body fat distribution,^9^ and, therefore, may not precisely estimate breast cancer risk among persons with excess adiposity. In an attempt to better capture the influence of body fat distribution on risk of breast cancer, several epidemiological studies have investigated the associations of waist circumference (WC) and waist‐to‐hip‐ratio (WHR),^4^, ^8^, ^10^, ^11^ indicators of central adiposity, with risk of postmenopausal breast cancer. In most studies, both measures have been positively associated with risk,^4^, ^8^, ^10^, ^12^ and some studies have suggested that these measures may better predict the risk of postmenopausal breast cancer than BMI.^4^, ^8^ BMI also fails to adequately account for differences in metabolic phenotypes among persons within the same BMI category.^13^, ^14^, ^15^ Consistent with these limitations, some women with normal BMI have enlarged adipocytes and elevated levels of leptin, suggestive of a hyperadipose state.^16^

Unlike anthropometric measures, dual‐energy X‐ray absorptiometry (DXA) provides estimates of fat, bone, and bone‐free lean mass comparable to those from more costly methods such as computed tomography and magnetic resonance imaging.^9^, ^17^ Using data from Women's Health Initiative (WHI), we have previously shown that various DXA‐derived measures of body fat were positively associated with risk of postmenopausal breast cancer.^18^ More recently, we also demonstrated that among normal BMI postmenopausal women, relatively high whole body fat and trunk fat (measured using DXA) were associated with almost twofold increases in risk of estrogen receptor‐positive (ER+) breast cancer.^19^

In contrast to anthropometric measures such as BMI, the use of more direct measures of body fat may more accurately determine the relationship between body fat distribution and risk of breast cancer risk among postmenopausal women. Thus, in the study reported here, which was conducted in a subset of participants in the WHI and represents an extension of an earlier report,^18^ we examined the association between DXA‐derived measures of body fat and risk of invasive breast cancer. We also evaluated whether the DXA‐derived measures influence risk of invasive breast cancer beyond that associated with the commonly used anthropometric measures and other conventional breast cancer risk factors.

## 2 METHODS

### 2.1 Study population and design

A detailed description of the WHI design and study population can be found elsewhere.^20^ Briefly, the WHI included 161 808 postmenopausal women aged 50 to 79, from major racial/ethnic groups, who were enrolled at 40 clinical centers throughout the United States between 1993 and 1998. Women were either included in the Clinical Trial (CT) group which has three overlapping components (hormone therapy (2 trials), low‐fat diet modification, and calcium‐vitamin D supplementation; n = 68 132) or the Observational Study (OS) group (n = 93 676).^20^ At baseline, information on demographic characteristics, menstrual history, reproductive history, exogenous hormone use, family history of breast cancer, medical history, and diet and lifestyle factors was collected, and anthropometric measurements (weight, height, waist circumference, hip circumference) were made by trained staff using a standardized protocol. After completion of the original study in 2005, WHI Extension Studies (2005‐2010, 2010‐2020) were conducted to gather additional follow‐up data. The study was approved by the institutional review boards of all participating institutions, and all participants provided written informed consent.

### 2.2 Body fat measurements

More detailed information on the ascertainment of DXA measures has been previously published.^21^ Briefly, for women at three WHI BMD clinical center (Birmingham, Tucson/Phoenix, and Pittsburgh; n = 11, 393), body fat was measured at baseline by whole body DXA scans performed in fan‐beam mode and obtained from Hologic QDR scanners (QDR 2000, 2000+, or 4500) (Hologic, Inc.). This subgroup of participants with DXA measures differed from those without DXA measures in that their recruitment was aimed at maximizing the number of ethnic/racial minority participants (22.6% non‐Hispanic white among those with DXA measures versus 16.9% among those without DXA measures).^22^ Scanners were operated by persons who were trained and certified on the basis of an evaluation of scanning and analysis technique. Scanner performance was monitored longitudinally using spine and whole body phantom scans.^21^ Quality control measures, including monitoring of phantom scans, reviewing random samples of all scans and flagging those with specific issues, controlling changes in hardware and software and scanning of calibration phantoms across instruments and clinical sites, were also implemented.^22^

Body fat measures included whole body fat (kg), percentage whole body fat, trunk fat (defined by the fat contained in the torso apart from head and limbs), fat mass of the legs, and the ratio of trunk to leg fat mass. In addition, the fat mass index (FMI) and trunk fat mass index (TFMI) were calculated as whole body fat mass and trunk fat mass in kilograms divided by height in meters squared, respectively.^23^, ^24^ The body fat to lean body mass ratio was also calculated as whole body fat mass divided by whole lean body mass. In addition to the baseline measures, the DXA scans and anthropometric assessments were repeated during four follow‐up visits (years 1, 3, 6, 9).

### 2.3 Analytic cohort

Our analytic cohort was restricted to women with available DXA measures (N = 11 393). Among these women, we excluded 108 women who were missing baseline DXA measures, 325 women with a previous history of breast cancer, and 29 women with missing information on follow‐up time. After exclusion, 10 931 women remained for our analyses, of whom 6078 were in the OS group and 4853 were in the CT intervention group (control, placebo, intervention) (See Figure S1).

### 2.4 Outcome ascertainment

Participants were followed up semi‐annually in the CT group and annually in the OS using in‐person, mailed, or telephone questionnaires to collect information on clinical outcomes. Breast cancer cases were confirmed via central review of medical records and pathology reports by trained physician adjudicators. The tumor hormone receptor status was coded using the National Cancer Institute's Surveillance Epidemiology and End Results (SEER) coding system.^25^ After a median follow‐up of 15.0 years (interquartile range: 8.8‐20.1 years), a total of 639 incident invasive breast cancer cases (including 484 ER+ breast cancer cases) had been ascertained.

### 2.5 Statistical analysis

Cox proportional hazards regression was used to estimate the age and multivariable adjusted hazard ratios (HRs) and 95% confidence intervals (CIs) for the associations between the body fat measures and risk of invasive breast cancer (overall and ER+) with time to diagnosis of invasive breast cancer as the underlying timescale. Participants were censored (non‐cases) if they died, withdrew from the study before the end of follow‐up, or did not develop invasive breast cancer by the end of follow‐up (February 28, 2017). Cases contributed person‐time to the study from their date of enrollment until the date of diagnosis of breast cancer, and non‐cases contributed person‐time from their date of enrollment until the date of death, the date of withdrawal from the study, or the end of follow‐up, whichever came first. For the analysis involving ER+ breast cancer, we additionally censored the non‐ ER+ breast cancer cases.

The body fat measures were analyzed as continuous variables (per standard deviation (SD) increase and per 5 or 2 kg increase) and as octiles. We used octiles in order to evaluate the full range of risk. Covariates were selected based on existing evidence or if they caused a 10% change in the HR estimates obtained from the multivariable models. For our main analyses, all regression models were adjusted for age at enrollment (continuous), education (high school or less, post‐secondary or some college, graduate school or some graduate school, missing), race/ethnicity (white, black, Hispanic, other), family history of breast cancer (yes, no), age (years) at menarche (>12, 12‐13, 14+), parity (never been pregnant or no term pregnancy, 1, 2, 3, 4+, missing), age (years) at menopause (>45, 45‐54, >55, missing), age (years) at first full‐term pregnancy (oral contraceptive use (yes, no), ever use of combined estrogen and progesterone therapy (yes, no), ever use of unopposed estrogen therapy (yes, no), physical activity (MET‐hours/week, continuous), alcohol intake ((servings/week), continuous), smoking (never, former, current), and study component (including OS group, and three CT group categories namely intervention, placebo, and control). Additional adjustment for diabetes and dietary calories from fat did not alter the estimates. Therefore, we did not adjust for these variables. We also performed similar analyses restricted to women who were not randomized to any of the clinical trials (ie, women from the observational study group). In three separate models, we additionally adjusted the DXA‐derived measures for BMI, WHR, or WC to assess whether the DXA‐derived measures influence risk of invasive breast cancer beyond that due to the anthropometric measures and other conventional breast cancer risk factors. To assess the nonlinear dose‐response relationship between the continuous body fat measures and risk of ER+ breast cancer, restricted cubic splines with three knots at the 25th, 50th, and 75th percentiles were fitted in Cox proportional hazards regression models.^26^ A *P* value for non‐linearity was determined by testing whether the coefficient of the second spline transformation was equal to zero (*P* < .05). In sensitivity analyses, we excluded women with a breast cancer diagnosis within two years of enrollment to assess for possible reverse causation.

*P*‐values for trend (*P*‐trend) were determined by including the ordinal body fat variable as continuous variables in the regression models and using Wald tests to assess statistical significance. The proportional hazards assumption was confirmed using Schoenfeld residuals.

We also examined the associations between the DXA‐derived measures and risk of invasive breast cancer by hormone therapy (HT) status (never, former, current) to assess whether HT use at baseline is an effect modifier. For this analysis, the exposures were categorized by quintiles, given the relatively small number of cases in some strata. Also, women in the HT intervention arms (ie, estrogen alone intervention, estrogen and progesterone intervention) were excluded. To estimate the P‐value for heterogeneity, we included an interaction term in the Cox regression models and used the Wald test to test its coefficient.

To account for the influence of changes in the measurements over time on risk of breast cancer, Cox proportional hazards regression models with time‐dependent exposures were also created. We conducted sensitivity analyses whereby physical activity, alcohol consumption, and smoking were also included as time‐varying covariates. However, the estimates were virtually unchanged. Therefore, only the body fat measures were fitted as time‐dependent. These models were adjusted for the aforementioned covariates (except the anthropometric measures).

All statistical analyses were performed using Stata 14.1 (StataCorp). *P*‐values were two‐sided. *P*‐values were considered to be statistically significant if <.05.

## 3 RESULTS

Table 1 presents a summary of the participants' characteristics by octiles of trunk fat mass. Among women with trunk fat mass in the highest octile, mean levels of BMI, WC, and WHR were highest while mean physical activity level and alcohol intake were lowest. Women with elevated trunk fat mass levels (highest octile) were also less likely to be HT‐users at baseline but were more likely to have started menstruating at an early age (<12).

**Table 1:** Distribution of selected characteristics by octiles of trunk fat mass

	Octiles of trunk fat mass								*P*‐value
	1	2	3	4	5	6	7	8	
Age (years), mean	63.2	63.7	63.9	63.7	64.2	63.4	62.7	61.2	<.001
Family history of breast cancer (%)	16.8	14.8	16.8	16.4	16.8	16.3	15.2	15.8	.737
Age at menarche (% <12 y)	16.1	15.5	20.1	20.5	21.1	22.0	25.3	27.8	<.001
Age at first full‐term pregnancy (% ≥30 y)	7.5	6.1	7.1	5.5	5.6	5.3	6.3	6.1	<.001
Parity (% Nulliparous)	12.5	11.6	12.2	11.6	8.9	9.2	10.0	10.4	<.001
Age at menopause (% ≥55 y)	8.6	9.1	6.7	7.8	6.9	7.2	7.7	6.3	<.001
Body mass index (kg/m^2^), mean	21.4	23.6	25.2	26.6	28.2	30.0	32.7	38.0	<.001
Waist circumference (cm), mean	69.2	74.6	78.8	82.6	86.4	90.8	96.8	107.0	<.001
Waist to hip ratio, mean	0.74	0.77	0.79	0.80	0.82	0.83	0.84	0.85	<.001
Oral contraceptive use (%)	38.6	37.2	36.4	36.1	36.7	36.6	37.0	39.4	.595
HT use (%)	57.8	60.4	57.0	53.9	54.3	47.8	49.1	43.5	<.001
Alcohol consumption (servings/week), mean	2.3	2.1	2.1	1.8	1.8	1.4	1.2	1.0	<.001
Physical activity (MET‐hours/week), mean	16.6	13.1	11.2	10.9	9.5	7.9	7.1	5.7	<.001
Smoking (never; %)	52.5	56.2	53.3	55.6	55.9	56.6	56.1	53.3	.106
Education (% post‐college)	31.2	25.6	22.6	20.7	20.1	19.6	19.5	17.1	<.001
Race/ethnicity (%)									
Non‐Hispanic white	88.4	84.1	80.2	79.5	78.2	75.0	70.0	67.5	<.001
Black	6.2	9.1	11.6	12.4	13.3	14.8	20.2	23.4	
Hispanic	3.4	5.0	6.7	6.5	6.8	8.0	6.8	6.4	
Other	2.0	1.8	1.5	1.6	1.7	2.3	3.0	2.7	

In multivariable analyses (excluding BMI, WC or WHR), we observed that all DXA‐derived measures were positively associated with risk of invasive breast cancer (Table 2; Figure S2). In particular, whole body fat mass, trunk fat mass, and the body fat indices (ie, FMI and TFMI) in the highest octile had more than a doubling of the risk of invasive breast cancer compared to women in the lowest octile (HR: 2.13, 95% CI: 1.52‐2.98; 2.58, 1.83‐3.65; 2.14, 1.53‐3.01, and 2.17, 1.52‐3.07, respectively) (Table 2).

**Table 2:** Hazard ratios and 95% CI for the associations of baseline DXA‐derived body fat measures and incident, invasive breast cancer in postmenopausal women

	Cases/person‐years	Age‐adjusted HR (95% CI)^a^	Multivariable‐adjusted HR (95% CI)^b^	Multivariable‐adjusted HR (95% CI)^c^	Multivariable‐adjusted HR (95% CI)^d^	Multivariable‐adjusted HR (95% CI)^e^
Whole body fat mass (kg)						
Per SD increase		1.18 (1.09‐1.27)	1.21 (1.12‐1.30)	1.18 (0.98‐1.41)	1.11 (0.97‐1.28)	1.18 (1.09‐1.28)
Octiles^f^						
1	57/19708.8	1.00	1.00	1.00	1.00	1.00
2	65/19867.8	1.13 (0.79‐1.62)	1.19 (0.83‐1.70)	1.18 (0.82‐1.69)	1.17 (0.81‐1.66)	1.17 (0.82‐1.67)
3	76/19672.6	1.34 (0.95‐1.88)	1.42 (1.01‐2.01)	1.39 (0.97‐2.00)	1.37 (0.96‐1.96)	1.38 (0.97‐1.95)
4	73/19883.7	1.27 (0.90‐1.80)	1.37 (0.97‐1.94)	1.37 (0.94‐2.00)	1.30 (0.90‐1.89)	1.31 (0.92‐1.87)
5	95/20054.6	1.64 (1.18‐2.28)	1.82 (1.31‐2.54)	1.83 (1.25‐2.67)	1.71 (1.18‐2.48)	1.73 (1.23‐2.42)
6	81/19388.8	1.45 (1.03‐2.04)	1.60 (1.13‐2.25)	1.60 (1.05‐2.42)	1.45 (0.97‐2.17)	1.48 (1.04‐2.10)
7	92/19189.0	1.67 (1.20‐2.33)	1.85 (1.32‐2.59)	1.85 (1.17‐2.92)	1.66 (1.08‐2.54)	1.72 (1.21‐2.43)
8	100/18698.0	1.89 (1.36‐2.62)	2.13 (1.52‐2.98)	2.12 (1.20‐3.79)	1.83 (1.11‐3.02)	1.98 (1.40‐2.80)
*P* for trend		<.001	<.001	.004	.014	<.001
Whole body fat percent						
Per SD increase		1.12 (1.04‐1.22)	1.16 (1.07‐1.26)	1.04 (0.92‐1.17)	1.04 (0.93‐1.16)	1.13 (1.04‐1.23)
Octiles^f^						
1	63/20332.0	1.00	1.00	1.00	1.00	1.00
2	67/19801.3	1.09 (0.77‐1.54)	1.14 (0.81‐1.61)	1.08 (0.76‐1.53)	1.07 (0.76‐1.52)	1.12 (0.79‐1.58)
3	85/20265.4	1.35 (0.98‐1.88)	1.47 (1.06‐2.04)	1.33 (0.95‐1.86)	1.31 (0.94‐1.83)	1.40 (1.01‐1.95)
4	82/20071.8	1.32 (0.95‐1.83)	1.45 (1.04‐2.02)	1.28 (0.90‐1.81)	1.24 (0.88‐1.75)	1.37 (0.98‐1.91)
5	87/19718.9	1.42 (1.03‐1.97)	1.57 (1.13‐2.18)	1.33 (0.94‐1.90)	1.29 (0.91‐1.84)	1.46 (1.05‐2.05)
6	79/18786.2	1.36 (0.98‐1.90)	1.51 (1.08‐2.12)	1.22 (0.84‐1.77)	1.20 (0.83‐1.73)	1.40 (1.00‐1.98)
7	97/19058.1	1.65 (1.20‐2.26)	1.83 (1.32‐2.53)	1.40 (0.96‐2.05)	1.39 (0.96‐2.01)	1.69 (1.22‐2.36)
8	79/18429.5	1.39 (1.00‐1.94)	1.57 (1.12‐2.20)	1.06 (0.67‐1.65)	1.09 (0.72‐1.65)	1.47 (1.04‐2.07)
*P* for trend		.003	<.001	.373	.420	.003
Trunk fat mass (kg)						
Per SD increase		1.18 (1.09‐1.27)	1.21 (1.12‐1.31)	1.19 (1.02‐1.40)	1.13 (0.97‐1.33)	1.18 (1.09‐1.29)
Octiles^f^						
1	51/20173.2	1.00	1.00	1.00	1.00	1.00
2	78/20018.3	1.54 (1.08‐2.19)	1.64 (1.15‐2.33)	1.62 (1.13‐2.33)	1.62 (1.13‐2.32)	1.61 (1.13‐2.30)
3	80/19814.0	1.59 (1.12‐2.26)	1.74 (1.22‐2.47)	1.70 (1.18‐2.46)	1.71 (1.18‐2.47)	1.69 (1.18‐2.41)
4	62/19722.3	1.24 (0.86‐1.80)	1.37 (0.94‐1.99)	1.38 (0.93‐2.05)	1.34 (0.89‐2.00)	1.31 (0.90‐1.92)
5	95/19994.8	1.87 (1.33‐2.63)	2.12 (1.50‐2.99)	2.15 (1.46‐3.15)	2.05 (1.38‐3.04)	2.01 (1.41‐2.87)
6	89/19243.0	1.83 (1.30‐2.58)	2.09 (1.47‐2.97)	2.13 (1.41‐3.22)	2.01 (1.31‐3.08)	1.97 (1.37‐2.84)
7	80/18792.0	1.69 (1.19‐2.41)	1.92 (1.34‐2.74)	1.96 (1.24‐3.08)	1.80 (1.12‐2.87)	1.76 (1.21‐2.57)
8	104/18705.7	2.23 (1.60‐3.13)	2.58 (1.83‐3.65)	2.67 (1.56‐4.57)	2.41 (1.40‐4.16)	2.41 (1.67‐3.47)
*P* for trend		<.001	<.001	.002	.012	<.001
Fat mass of right leg (kg)						
Per SD increase		1.15 (1.07‐1.24)	1.16 (1.08‐1.25)	1.05 (0.93‐1.18)	1.07 (0.97‐1.18)	1.16 (1.08‐1.25)
Octiles^f^						
1	55/18797.8	1.00	1.00	1.00	1.00	1.00
2	75/19409.6	1.33 (0.94‐1.88)	1.35 (0.95‐1.91)	1.28 (0.90‐1.82)	1.30 (0.92‐1.85)	1.35 (0.95‐1.91)
3	71/19702.3	1.24 (0.87‐1.77)	1.27 (0.89‐1.80)	1.20 (0.83‐1.71)	1.18 (0.83‐1.69)	1.24 (0.87‐1.77)
4	80/19992.4	1.38 (0.98‐1.95)	1.42 (1.00‐2.00)	1.33 (0.94‐1.90)	1.32 (0.93‐1.87)	1.42 (1.00‐2.00)
5	79/20047.8	1.36 (0.97‐1.92)	1.41 (1.00‐2.00)	1.30 (0.91‐1.87)	1.28 (0.90‐1.83)	1.40 (0.99‐1.98)
6	81/19433.4	1.44 (1.02‐2.03)	1.49 (1.06‐2.11)	1.35 (0.93‐1.95)	1.32 (0.92‐1.89)	1.47 (1.04‐2.08)
7	92/20249.2	1.57 (1.13‐2.20)	1.65 (1.18‐2.32)	1.45 (0.99‐2.11)	1.42 (0.99‐2.04)	1.64 (1.17‐2.30)
8	106/18830.8	1.98 (1.42‐2.74)	2.05 (1.47‐2.87)	1.65 (1.07‐2.55)	1.63 (1.11‐2.39)	2.03 (1.45‐2.83)
*P* for trend		<.001	<.001	.051	.033	<.001
Fat mass of left leg (kg)						
Per SD increase		1.15 (1.06‐1.24)	1.16 (1.07‐1.25)	1.04 (0.92‐1.17)	1.06 (0.96‐1.17)	1.16 (1.07‐1.25)
Octiles^f^						
1	57/18976.4	1.00	1.00	1.00	1.00	1.00
2	74/19360.2	1.28 (0.91‐1.81)	1.31 (0.92‐1.85)	1.24 (0.88‐1.76)	1.25 (0.88‐1.77)	1.30 (0.92‐1.84)
3	72/19605.3	1.23 (0.87‐1.74)	1.26 (0.89‐1.79)	1.15 (0.81‐1.64)	1.17 (0.82‐1.66)	1.24 (0.87‐1.76)
4	77/20000.1	1.29 (0.92‐1.82)	1.34 (0.95‐1.89)	1.22 (0.86‐1.74)	1.23 (0.87‐1.74)	1.34 (0.95‐1.89)
5	87/19877.7	1.47 (1.05‐2.06)	1.53 (1.09‐2.14)	1.36 (0.95‐1.93)	1.35 (0.96‐1.91)	1.50 (1.07‐2.10)
6	81/19865.6	1.37 (0.98‐1.92)	1.43 (1.01‐2.01)	1.22 (0.85‐1.77)	1.24 (0.87‐1.76)	1.41 (1.00‐1.98)
7	93/20007.7	1.57 (1.13‐2.18)	1.63 (1.17‐2.28)	1.35 (0.93‐1.96)	1.36 (0.95‐1.94)	1.62 (1.16‐2.26)
8	98/18770.2	1.78 (1.28‐2.47)	1.85 (1.32‐2.58)	1.34 (0.87‐2.07)	1.39 (0.95‐2.05)	1.82 (1.30‐2.54)
*P* for trend		<.001	<.001	.191	.119	<.001
Ratio of trunk fat mass to average of R and L leg fat mass						
Octiles^f^						
1	58/20171.1	1.00	1.00	1.00	1.00	1.00
2	78/20416.0	1.33 (0.94‐1.86)	1.39 (0.99‐1.95)	1.29 (0.91‐1.82)	1.27 (0.90‐1.80)	1.34 (0.95‐1.88)
3	98/20251.7	1.68 (1.22‐2.33)	1.79 (1.29‐2.48)	1.58 (1.14‐2.21)	1.56 (1.12‐2.18)	1.68 (1.21‐2.35)
4	89/19707.1	1.57 (1.13‐2.19)	1.68 (1.20‐2.35)	1.46 (1.03‐2.05)	1.43 (1.01‐2.02)	1.56 (1.11‐2.20)
5	74/19678.6	1.30 (0.92‐1.84)	1.44 (1.01‐2.03)	1.23 (0.86‐1.77)	1.19 (0.83‐1.71)	1.30 (0.91‐1.87)
6	81/19144.2	1.47 (1.05‐2.06)	1.63 (1.16‐2.29)	1.41 (0.99‐2.00)	1.32 (0.92‐1.90)	1.46 (1.02‐2.08)
7	71/18960.8	1.30 (0.92‐1.84)	1.45 (1.02‐2.07)	1.23 (0.85‐1.77)	1.14 (0.78‐1.66)	1.27 (0.87‐1.84)
8	90/18133.7	1.73 (1.24‐2.40)	1.96 (1.40‐2.75)	1.66 (1.17‐2.36)	1.46 (1.01‐2.13)	1.63 (1.12‐2.37)
*P* for trend		.052	.006	.142	.639	.251
Fat mass index (kg/m^2^)						
Per SD increase		1.15 (1.07‐1.23)	1.18 (1.09‐1.27)	1.05 (0.86‐1.29)	1.05 (0.92‐1.19)	1.15 (1.06‐1.25)
Octiles^f^						
1	57/19857.0	1.00	1.00	1.00	1.00	1.00
2	77/20212.9	1.32 (0.94‐1.87)	1.41 (1.00‐1.98)	1.37 (0.97‐1.95)	1.36 (0.96‐1.92)	1.38 (0.98‐1.95)
3	66/19535.3	1.18 (0.83‐1.68)	1.27 (0.89‐1.82)	1.22 (0.84‐1.77)	1.19 (0.82‐1.72)	1.22 (0.86‐1.75)
4	84/19950.1	1.46 (1.04‐2.05)	1.62 (1.15‐2.28)	1.52 (1.05‐2.22)	1.48 (1.03‐2.13)	1.55 (1.10‐2.18)
5	85/19672.5	1.50 (1.07‐2.10)	1.70 (1.21‐2.39)	1.57 (1.06‐2.34)	1.51 (1.03‐2.20)	1.59 (1.12‐2.25)
6	85/19124.9	1.56 (1.11‐2.18)	1.72 (1.22‐2.42)	1.55 (1.01‐2.40)	1.47 (0.99‐2.25)	1.58 (1.12‐2.25)
7	85/19092.4	1.56 (1.11‐2.18)	1.77 (1.25‐2.50)	1.56 (0.96‐2.53)	1.47 (0.96‐2.25)	1.63 (1.15‐2.33)
8	98/18366.8	1.88 (1.36‐2.61)	2.14 (1.53‐3.01)	1.75 (0.94‐3.27)	1.66 (1.02‐2.70)	1.98 (1.40‐2.81)
*P* for trend		<.001	<.001	.110	.084	<.001
Trunk fat mass index (kg/m^2^)						
Per SD increase		1.15 (1.07‐1.24)	1.19 (1.10‐1.28)	1.12 (0.94‐1.34)	1.06 (0.92‐1.23)	1.16 (1.06‐1.26)
Octiles^f^						
1	54/20288.3	1.00	1.00	1.00	1.00	1.00
2	89/ 20 443.5	1.63 (1.16‐2.28)	1.69 (1.20‐2.38)	1.65 (1.16‐2.33)	1.63 (1.15‐2.29)	1.66 (1.18‐2.33)
3	66/19922.2	1.24 (0.87‐1.78)	1.34 (0.93‐1.93)	1.29 (0.88‐1.88)	1.25 (0.86‐1.83)	1.30 (0.90‐1.87)
4	72/19802.3	1.36 (0.96‐1.94)	1.51 (1.05‐2.15)	1.42 (0.97‐2.08)	1.37 (0.93‐2.01)	1.43 (0.99‐2.06)
5	84/ 19 899.6	1.58 (1.12‐2.22)	1.77 (1.25‐2.51)	1.64 (1.11‐2.43)	1.57 (1.07‐2.33)	1.67 (1.17‐2.39)
6	84/19249.9	1.63 (1.16‐2.30)	1.86 (1.31‐2.63)	1.68 (1.11‐2.56)	1.59 (1.05‐2.41)	1.72 (1.20‐2.47)
7	96/ 18 927.9	1.91 (1.37‐2.67)	2.18 (1.55‐3.07)	1.90 (1.21‐3.00)	1.77 (1.14‐2.77)	1.98 (1.38‐2.84)
8	92/18508.0	1.89 (1.35‐2.65)	2.17 (1.52‐3.07)	1.80 (1.02‐3.19)	1.66 (0.98‐2.82)	1.99 (1.38‐2.87)
*P* for trend		<.001	<.001	.064	.110	<.001

a Adjusted for age at enrollment

b Adjusted for age at enrollment, education, race/ethnicity, family history of breast cancer, age at menarche, age at first full‐term birth, parity, age at menopause, oral contraceptive use, hormone therapy use, physical activity, alcohol intake, smoking, and study component

c Also adjusted for BMI

d Also adjusted for waist circumference

e Also adjusted for waist to hip ratio

f Cut‐points‐ whole body fat mass (kg):‐ ≤20.39, 27.40‐24.32, 24.33‐27.72, 27.73‐30.90, 30.91‐34.53, 34.54‐38.90, 38.91‐45.93, >45.93; whole body percent fat (kg): ≤35.5, 35.6‐39.3, 39.4‐42.0, 42.1‐44.4, 44.5‐46.6, 46.7‐49.0, 49.1‐52.0, >52, trunk fat mass (kg): ≤8.23, 8.24‐10.65, 10.66‐12.62, 12.63‐14.43, 14.44‐16.44, 16.45‐18.85, 18.86‐22.33, >22.33; fat mass of right leg (kg): <3.98, 3.99‐4.66, 4.67‐5.23, 5.24‐5.81, 5.82‐6.46, 6.47‐7.24, 7.25‐8.57, >8.57; fat mass of left leg (kg): <3.87, 3.88‐4.54, 4.55‐5.09, 5.10‐5.67, 5.68‐6.31, 6.32‐7.08, 7.08‐8.39, >8.39, ratio of trunk fat mass to average of R and L leg fat mass: ≤1.66, 1.66‐1.98, 1.99‐2.21, 2.22‐2.43, 2.43‐2.68, 2.68‐2.96, 2.97‐3.39, >3.39; body fat index:‐ ≤7.85, 7.85‐9.34, 9.35‐10.64, 10.65‐11.87, 11.88‐13.29, 13.30‐14.91, 14.92‐17.54, >17.54; trunk fat mass index: ≤3.14, 3.15‐4.09, 4.10‐4.85, 4.86‐ 5.57, 5.58‐6.31, 6.32‐7.20, 7.20‐8.58, >8.58 for octiles 1, 2, 3, 4, 5, 6, 7 and 8, respectively. Two cases had missing values for the body fat index and the trunk fat mass index.

The multivariable models (excluding adjustment for whole body fat mass or trunk fat mass) also demonstrated positive associations of all of the anthropometric measures (examined as categorical variables) with risk of invasive breast cancer overall and with ER+ breast cancer, but these associations did not persist after further adjustment for whole body fat mass or trunk fat mass (Tables S1‐S2). When considering the continuous exposures, the HRs per SD increase in the anthropometric measures (Tables S1‐S2) were of similar magnitude to those seen for the DXA‐derived measures (Tables 2,3).

**Table 3:** Hazard ratios and 95% CI for the association of baseline DXA‐derived body fat measures and incident, ER‐positive breast cancer in postmenopausal women

	Cases/person‐years	Age‐adjusted HR (95% CI)^a^	Multivariable‐adjusted HR (95% CI)^b^	Multivariable‐adjusted HR (95% CI)^c^	Multivariable‐adjusted HR (95% CI)^d^	Multivariable‐adjusted HR (95% CI)^e^
Whole body fat mass (kg)						
Per SD increase		1.16 (1.06‐1.26)	1.22 (1.11‐1.33)	1.28 (1.02‐1.59)	1.18 (1.00‐1.39)	1.19 (1.08‐1.31)
Octiles^f^						
1	46/19708.8	1.00	1.00	1.00	1.00	1.00
2	48/19867.8	1.04 (0.69‐1.55)	1.12 (0.75‐1.68)	1.11 (0.73‐1.68)	1.11 (0.74‐1.68)	1.10 (0.73‐1.66)
3	55/19672.6	1.20 (0.81‐1.71)	1.32 (0.86‐1.96)	1.35 (0.89‐2.05)	1.31 (0.87‐1.97)	1.29 (0.87‐1.91)
4	58/19883.7	1.25 (0.85‐1.84)	1.40 (0.95‐2.07)	1.43 (0.94‐2.19)	1.38 (0.91‐2.09)	1.35 (0.91‐1.99)
5	76/20054.6	1.63 (1.13‐2.35)	1.90 (1.31‐2.75)	1.96 (1.28‐3.02)	1.86 (1.22‐2.83)	1.81 (1.24‐2.64)
6	66/19388.8	1.47 (1.01‐2.14)	1.72 (1.17‐2.52)	1.79 (1.11‐2.87)	1.65 (1.04‐2.60)	1.60 (1.08‐2.37)
7	65/19189.0	1.48 (1.01‐2.15)	1.75 (1.19‐2.57)	1.84 (1.08‐3.14)	1.69 (1.03‐2.77)	1.64 (1.10‐2.44)
8	70/18698.0	1.67 (1.15‐2.42)	2.06 (1.40‐3.02)	2.22 (1.14‐4.35)	1.96 (1.10‐3.50)	1.93 (1.30‐2.86)
*P* for trend		<.001	<.001	.003	.007	<.001
Whole body fat percent						
Per SD increase		1.13 (1.03‐1.24)	1.19 (1.09‐1.31)	1.12 (0.97‐1.29)	1.11 (0.98‐1.26)	1.17 (1.06‐1.29)
Octiles^f^						
1	48/20332.0	1.00	1.00	1.00	1.00	1.00
2	51/19801.3	1.09 (0.74‐1.62)	1.17 (0.78‐1.73)	1.10 (0.74‐1.64)	1.12 (0.75‐1.66)	1.14 (0.77‐1.70)
3	66/20265.4	1.38 (0.95‐2.00)	1.55 (1.07‐2.26)	1.44 (0.98‐2.12)	1.43 (0.97‐2.10)	1.49 (1.02‐2.17)
4	61/20071.8	1.29 (0.88‐1.88)	1.48 (1.01‐2.16)	1.33 (0.89‐1.99)	1.32 (0.88‐1.96)	1.40 (0.95‐2.06)
5	68/19718.9	1.46 (1.01‐2.11)	1.72 (1.18‐2.50)	1.51 (1.01‐2.26)	1.49 (0.99‐2.22)	1.60 (1.10‐2.35)
6	61/18786.2	1.38 (0.95‐2.02)	1.62 (1.10‐2.38)	1.38 (0.90‐2.12)	1.37 (0.90‐2.09)	1.51 (1.02‐2.24)
7	66/19058.1	1.47 (1.01‐2.13)	1.75 (1.19‐2.56)	1.44 (0.93‐2.21)	1.36 (0.88‐2.11)	1.63 (1.11‐2.40)
8	63/18429.5	1.47 (1.01‐2.15)	1.80 (1.23‐2.65)	1.39 (0.86‐2.23)	1.26 (0.78‐2.03)	1.70 (1.15‐2.51)
*P* for trend		.019	.001	.275	.446	.012
Trunk fat mass (kg)						
Per SD increase		1.15 (1.05‐1.25)	1.21 (1.11‐1.33)	1.22 (1.01‐1.47)	1.18 (0.98‐1.42)	1.18 (1.07‐1.31)
Octiles^f^						
1	40/20173.2	1.00	1.00	1.00	1.00	1.00
2	60/20018.3	1.51 (1.01‐2.25)	1.65 (1.10‐2.46)	1.66 (1.10‐2.49)	1.64 (1.09‐2.47)	1.63 (1.09‐2.44)
3	61/19814.0	1.55 (1.04‐2.31)	1.76 (1.18‐2.63)	1.74 (1.15‐2.64)	1.76 (1.15‐2.68)	1.72 (1.15‐2.58)
4	46/19722.3	1.17 (0.77‐1.79)	1.35 (0.88‐2.08)	1.36 (0.86‐2.14)	1.35 (0.85‐2.14)	1.31 (0.85‐2.02)
5	78/19994.8	1.95 (1.33‐2.86)	2.32 (1.58‐3.42)	2.35 (1.52‐3.62)	2.32 (1.48‐3.62)	2.23 (1.50‐3.32)
6	68/19243.0	1.79 (1.21‐2.64)	2.18 (1.46‐3.25)	2.21 (1.38‐3.54)	2.16 (1.33‐3.53)	2.07 (1.37‐3.13)
7	61/18792.0	1.65 (1.11‐2.47)	2.02 (1.35‐3.04)	2.06 (1.22‐3.46)	1.97 (1.15‐3.39)	1.88 (1.22‐2.88)
8	70/18705.7	1.95 (1.32‐2.87)	2.45 (1.64‐3.66)	2.51 (1.34‐4.66)	2.43 (1.29‐4.59)	2.31 (1.51‐3.53)
*P* for trend		.001	<.001	.005	.011	<.001
Fat mass of right leg (kg)						
Per SD increase		1.15 (1.06‐1.26)	1.19 (1.09‐1.30)	1.12 (0.98‐1.29)	1.12 (1.00‐1.25)	1.19 (1.09‐1.30)
Octiles^f^						
1	42/18797.8	1.00	1.00	1.00	1.00	1.00
2	56/19409.6	1.30 (0.87‐1.94)	1.33 (0.89‐1.98)	1.27 (0.85‐1.91)	1.29 (0.86‐1.93)	1.33 (0.89‐1.99)
3	49/19702.3	1.13 (0.75‐1.70)	1.15 (0.76‐1.74)	1.11 (0.73‐1.68)	1.07 (0.71‐1.63)	1.12 (0.74‐1.69)
4	63/19992.4	1.43 (0.97‐2.11)	1.49 (1.01‐2.21)	1.41 (0.94‐2.12)	1.40 (0.94‐2.09)	1.49 (1.00‐2.20)
5	65/20047.8	1.47 (0.99‐2.17)	1.58 (1.07‐2.33)	1.47 (0.98‐2.22)	1.46 (0.98‐2.18)	1.56 (1.06‐2.31)
6	64/19433.4	1.50 (1.02‐2.22)	1.60 (1.08‐2. 36)	1.46 (0.96‐2.23)	1.44 (0.96‐2.18)	1.58 (1.06‐2.33)
7	73/20249.2	1.64 (1.12‐2.41)	1.80 (1.22‐2.64)	1.61 (1.04‐2.48)	1.58 (1.05‐2.39)	1.78 (1.21‐2.62)
8	72/18830.8	1.79 (1.22‐2.62)	1.99 (1.34‐2.93)	1.65 (0.99‐2.75)	1.64 (1.05‐2.57)	1.95 (1.32‐2.89)
*P *for trend		.001	<.001	.022	.013	<.001
Fat mass of left leg (kg)						
Per SD increase		1.14 (1.05‐1.25)	1.18 (1.08‐1.29)	1.09 (0.95‐1.26)	1.10 (0.99‐1.24)	1.18 (1.08‐1.29)
Octiles^f^						
1	45/18976.4	1.00	1.00	1.00	1.00	1.00
2	53/19360.2	1.16 (0.78‐1.73)	1.19 (0.80‐1.78)	1.13 (0.76‐1.69)	1.15 (0.77‐1.71)	1.19 (0.80‐1.77)
3	53/19605.3	1.15 (0.77‐1.71)	1.18 (0.79‐1.76)	1.12 (0.75‐1.67)	1.10 (0.74‐1.65)	1.16 (0.78‐1.73)
4	60/20000.1	1.28 (0.87‐1.89)	1.35 (0.92‐2.00)	1.24 (0.83‐1.85)	1.25 (0.85‐1.86)	1.35 (0.92‐2.00)
5	70/19877.7	1.51 (1.04‐2.19)	1.61 (1.10‐2.34)	1.44 (0.97‐2.13)	1.45 (0.98‐2.14)	1.58 (1.08‐2.30)
6	66/19865.6	1.42 (0.97‐2.08)	1.53 (1.04‐2.24)	1.32 (0.88‐2.00)	1.35 (0.90‐2.01)	1.51 (1.03‐2.21)
7	70/20007.7	1.50 (1.03‐2.19)	1.63 (1.12‐2.39)	1.36 (0.89‐2.08)	1.39 (0.93‐2.09)	1.62 (1.11‐2.37)
8	67/18770.2	1.57 (1.07‐2.29)	1.73 (1.18‐2.55)	1.28 (0.78‐2.12)	1.35 (0.86‐2.12)	1.70 (1.16‐2.51)
*P *for trend		.003	.001	.123	.070	.001
Ratio of trunk fat mass to average of R and L leg fat mass						
Octiles^f^						
1	45/20171.1	1.00	1.00	1.00	1.00	1.00
2	56/20416.0	1.22 (0.83‐1.81)	1.31 (0.88‐1.95)	1.23 (0.82‐1.83)	1.21 (0.81‐1.81)	1.26 (0.85‐1.88)
3	82/20251.7	1.81 (1.26‐2.60)	1.97 (1.37‐2.84)	1.78 (1.23‐2.59)	1.74 (1.20‐2.54)	1.86 (1.28‐2.70)
4	69/19707.1	1.57 (1.08‐2.29)	1.76 (1.20‐2.57)	1.55 (1.05‐2.30)	1.51 (1.02‐2.24)	1.63 (1.11‐2.41)
5	52/19678.6	1.18 (0.79‐1.75)	1.35 (0.90‐2.01)	1.16 (0.76‐1.76)	1.13 (0.74‐1.72)	1.22 (0.81‐1.85)
6	64/19144.2	1.50 (1.02‐2.19)	1.72 (1.17‐2.54)	1.50 (1.01‐2.24)	1.41 (0.94‐2.14)	1.54 (1.03‐2.31)
7	50/18960.8	1.18 (0.79‐1.76)	1.37 (0.91‐2.07)	1.17 (0.77‐1.79)	1.09 (0.70‐1.69)	1.20 (0.78‐1.85)
8	66/18133.7	1.64 (1.12‐2.39)	1.95 (1.32‐2.88)	1.67 (1.12‐2.51)	1.48 (0.96‐2.28)	1.61 (1.05‐2.49)
*P *for trend		.218	.150	.271	.767	.406
Fat mass index (kg/m^2^)						
Continuous per SD increase		1.12 (1.03‐1.22)	1.19 (1.09‐1.29)	1.20 (0.92‐1.57)	1.10 (0.94‐1.28)	1.16 (1.06‐1.27)
Octiles^f^						
1	44/19857.0	1.00	1.00	1.00	1.00	1.00
2	58/20212.9	1.29 (0.87‐1.91)	1.41 (0.95‐2.09)	1.38 (0.93‐2.07)	1.37 (0.92‐2.05)	1.39 (0.94‐2.06)
3	52/19535.3	1.20 (0.81‐1.80)	1.35 (0.90‐2.02)	1.31 (0.85‐2.01)	1.28 (0.84‐1.95)	1.31 (0.87‐1.96)
4	62/19950.1	1.39 (0.95‐2.05)	1.62 (1.10‐2.40)	1.56 (1.01‐2.41)	1.52 (1.00‐2.31)	1.56 (1.05‐2.32)
5	72/19672.5	1.65 (1.13‐2.40)	1.97 (1.35‐2.89)	1.87 (1.19‐2.95)	1.81 (1.18‐2.77)	1.87 (1.27‐2.75)
6	65/19124.9	1.54 (1.05‐2.25)	1.83 (1.24‐2.70)	1.71 1.03‐2.84)	1.62 (1.03‐2.56)	1.70 (1.14‐2.54)
7	65/19092.4	1.55 (1.06‐2.27)	1.93 (1.30‐2.85)	1.77 (1.01‐3.12)	1.68 (1.03‐2.75)	1.80 (1.20‐2.69)
8	65/18366.8	1.64 (1.12‐2.40)	2.05 (1.38‐3.05)	1.81 (0.87‐3.79)	1.71 (0.97‐3.02)	1.92 (1.28‐2.89)
*P *for trend		<.001	<.001	.064	.110	<.001
Trunk fat mass index (kg/m^2^)						
Continuous per SD increase		1.12 (1.03‐1.22)	1.19 (1.09‐1.30)	1.16 (0.95‐1.43)	1.09 (0.92‐1.29)	1.15 (1.05‐1.27)
Octiles^f^						
1	41/20108.9	1.00	1.00	1.00	1.00	1.00
2	68/20311.7	1.63 (1.11‐2.40)	1.74 (1.18‐2.57)	1.73 (1.17‐2.57)	1.71 (1.15‐2.54)	1.72 (1.16‐2.54)
3	50/19761.5	1.24 (0.82‐1.87)	1.41 (0.93‐2.13)	1.39 (0.90‐2.15)	1.37 (0.88‐2.11)	1.37 (0.90‐2.08)
4	59/19662.3	1.46 (0.98‐2.18)	1.70 (1.14‐2.55)	1.68 (1.08‐2.60)	1.63 (1.05‐2.53)	1.64 (1.08‐2.47)
5	65/19761.0	1.60 (1.08‐2.36)	1.90 (1.28‐2.83)	1.87 (1.19‐2.95)	1.81 (1.15‐2.84)	1.82 (1.21‐2.74)
6	64/19142.1	1.64 (1.11‐2.43)	2.00 (1.34‐2.98)	1.95 (1.20‐3.18)	1.86 (1.15‐3.02)	1.88 (1.24‐2.85)
7	72/18744.9	1.90 (1.29‐2.78)	2.36 (1.59‐3.50)	2.30 (1.35‐3.91)	2.13 (1.27‐3.59)	2.18 (1.44‐3.29)
8	64/18319.4	1.75 (1.18‐2.60)	2.21 (1.47‐3.32)	2.13 (1.08‐4.19)	1.97 (1.06‐3.68)	2.07 (1.35‐3.18)
*P *for trend		<.001	<.001	.030	.054	.001

a Adjusted for age at enrollment

b Adjusted for age at enrollment, education, race/ethnicity, family history of breast cancer, age at menarche, age at first full‐term birth, parity, age at menopause, oral contraceptive use, hormone therapy use, physical activity, alcohol intake, smoking, and study component

c Also adjusted for BMI

d Also adjusted for waist circumference

e Also adjusted for waist to hip ratio

f Cut‐points‐ whole body fat mass (kg):‐ ≤20.39, 27.40‐24.32, 24.33‐27.72, 27.73‐30.90, 30.91‐34.53, 34.54‐38.90,38.91‐45.93, >45.93; whole body percent fat (kg): ≤35.5, 35.6‐39.3, 39.4‐42.0, 42.1‐44.4, 44.5‐46.6, 46.7‐49.0, 49.1‐52.0, >52, trunk fat mass (kg): ≤8.23, 8.24‐10.65, 10.66‐12.62, 12.63‐14.43, 14.44‐16.44, 16.45‐18.85, 18.86‐22.33,>22.33; fat mass of right leg (kg): <3.98, 3.99‐4.66, 4.67‐5.23, 5.24‐5.81, 5.82‐6.46, 6.47‐7.24, 7.25‐8.57, >8.57; fat mass of left leg (kg): <3.87, 3.88‐4.54, 4.55‐5.09, 5.10‐5.67, 5.68‐6.31, 6.32‐7.08, 7.08‐8.39, >8.39, ratio of trunk fat mass to average of R and L leg fat mass: ≤1.66, 1.66‐1.98, 1.99‐2.21, 2.22 ‐2.43, 2.43‐2.68, 2.68‐2.96,2.97‐3.39, >3.39, body fat index:‐ ≤7.85, 7.85‐9.34, 9.35‐10.64, 10.65‐11.87, 11.88‐13.29, 13.30‐14.91, 14.92‐17.54, >17.54; trunk fat mass index: ≤3.14, 3.15‐4.09, 4.10‐4.85, 4.86‐ 5.57, 5.58‐6.31, 6.32‐7.20, 7.20‐8.58, >8.58 for octiles 1, 2, 3, 4, 5, 6, 7 and 8, respectively. One case had a missing value for the body fat index and the trunk fat mass index.

To assess whether the DXA‐derived measures explained risk of breast cancer beyond that of the anthropometric measures and other conventional breast cancer risk factors, we additionally adjusted for the anthropometric measures (separately). In these analyses, the associations for whole body fat mass and trunk fat mass remained after further adjustment (separately) for BMI, WC, and WHR (Table 2). With respect to the body fat indices, the association for FMI disappeared after additional adjustment for BMI while, for TFMI, the association was no longer evident after additional adjustment for WC.

Table 4 and Figure S2 show that all DXA‐derived measures were positively associated with risk of ER+ breast cancer. Our multivariable analyses (excluding BMI, WC, and WHR) also demonstrated more than a twofold increased risk of ER+ breast cancer among women with whole body fat mass, trunk fat mass, FMI, and TFMI in the highest octile (HR: 2.06, 95% CI: 1.40‐3.02; 2.45, 1.64‐ 3.66; 2.05, 1.38‐3.05, and 2.21, 1.47‐3.32). Whole body fat mass, trunk fat mass, and TFMI remained independently associated with risk after additional adjustment for the anthropometric measures (separately) while the association for FMI only persisted after additional adjustment for WHR. The HRs per SD increase in the anthropometric measures were also of similar magnitude to those seen for the DXA‐derived measures (Tables 4 and Tables S2). The associations between the DXA‐derived measures and risk of ER+ breast cancer appeared to be nonlinear (Figure S3).

**Table 4:** Hazard ratios and 95% CI for the associations of baseline DXA‐derived body fat measures and incident, invasive breast cancer in postmenopausal women from the Observational Study group

	Overall	ER‐positive
	HR (95% CI)^a^	
Whole body fat mass (kg)		
Per SD increase	1.19 (1.07‐1.32)	1.20 (1.07‐1.36)
Octiles^b^		
1	1.00	1.00
2	1.09 (0.70‐1.70)	0.97 (0.59‐1.60)
3	1.57 (1.04‐2.37)	1.30 (0.81‐2.08)
4	1.24 (0.80‐1.93)	1.16 (0.71‐1.90)
5	1.89 (1.25‐2.86)	1.90 (1.21‐2.99)
6	1.39 (0.89‐2.19)	1.51 (0.93‐2.47)
7	1.88 (1.21‐2.92)	1.96 (1.21‐3.18)
8	1.96 (1.26‐3.05)	1.66 (0.99‐2.77)
*P* for trend	<.001	.002
Whole body fat percent		
Per SD increase	1.14 (1.02‐1.27)	1.18 (1.04‐1.34)
Octiles^b^		
1	1.00	1.00
2	0.99 (0.65‐1.54)	1.83 (0.83‐4.03)
3	1.52 (1.03‐2.25)	2.07 (0.95‐4.51)
4	1.35 (0.89‐2.04)	1.71 (0.77‐3.80)
5	1.46 (0.97‐2.21)	1.95 (0.89‐4.27)
6	1.17 (0.74‐1.84)	2.22 (1.03‐4.82)
7	1.81 (1.19‐2.73)	2.23 (1.03‐4.84)
8	1.36 (0.87‐2.13)	2.64 (1.22‐5.73)
*P* for trend	.010	.073
Trunk fat mass (kg)		
Per SD increase	1.19 (1.07‐1.32)	1.20 (1.06‐1.36)
Octiles^b^		
1	1.00	1.00
2	1.30 (0.85‐1.98)	1.41 (0.88‐2.26)
3	1.56 (1.03‐2.37)	1.48 (0.92‐2.41)
4	1.14 (0.72‐1.79)	0.96 (0.55‐1.66)
5	1.88 (1.24‐2.85)	2.28 (1.44‐3.60)
6	1.73 (1.11‐2.69)	1.82 (1.10‐3.01)
7	1.56 (0.98‐2.47)	1.85 (1.11‐3.09)
8	2.17 (1.40‐3.35)	2.06 (1.24‐3.44)
*P* for trend	<.001	.001
Fat mass of right leg (kg)		
Per SD increase	1.15 (1.04‐1.28)	1.18 (1.04‐1.33)
Octiles^b^		
1	1.00	1.00
2	1.47 (0.98‐2.24)	1.40 (0.87‐2.25)
3	1.11 (0.71‐1.76)	0.87 (0.51‐1.50)
4	1.39 (0.90‐2.15)	1.43 (0.88‐2.31)
5	1.36 (0.88‐2.12)	1.38 (0.85‐2.25)
6	1.49 (0.96‐2.33)	1.61 (0.98‐2.62)
7	1.63 (1.05‐2.53)	1.75 (1.08‐2.84)
8	1.92 (1.24‐2.98)	1.68 (1.00‐2.81)
*P* for trend	.003	.008
Fat mass of left leg (kg)		
Per SD increase	1.14 (1.03‐1.27)	1.17 (1.04‐1.32)
Octiles^b^		
1	1.00	1.00
2	1.38 (0.90‐2.11)	1.17 (0.72‐1.90)
3	1.18 (0.75‐1.85)	0.99 (0.59‐1.65)
4	1.26 (0.82‐1.96)	1.24 (0.77‐2.00)
5	1.60 (1.05‐2.45)	1.63 (1.02‐2.59)
6	1.40 (0.90‐2.17)	1.36 (0.84‐2.22)
7	1.60 (1.03‐2.47)	1.61 (0.99‐2.61)
8	1.72 (1.10‐2.98)	1.49 (0.89‐2.48)
*P *for trend	.001	.024
Ratio of trunk fat mass to average of R and L leg fat mass		
Octiles^b^		
1	1.00	1.00
2	1.31 (0.85‐2.00)	1.36 (0.84‐2.21)
3	1.73 (1.15‐2.60)	1.94 (1.22‐3.05)
4	1.56 (1.02‐2.39)	1.68 (1.03‐2.74)
5	1.37 (0.88‐2.13)	1.36 (0.82‐2.28)
6	1.73 (1.14‐2.64)	1.94 (1.21‐3.14)
7	1.19 (0.74‐1.90)	1.17 (0.68‐2.02)
8	1.81 (1.17‐2.81)	1.90 (1.15‐3.14)
*P* for trend	.432	.476
Fat mass index (kg/m^2^)		
Per SD increase	1.14 (1.03‐1.26))	1.15 (1.02‐1.29)
Octiles^b^		
1	1.00	1.00
2	1.25 (0.83‐1.91)	1.20 (0.75‐1.92)
3	1.23 (0.80‐1.88)	1.13 (0.70‐1.85)
4	1.63 (1.08‐2.47)	1.58 (0.99‐2.51)
5	1.48 (0.96‐2.27)	1.58 (0.98‐2.53)
6	1.54 (0.99‐2.38)	1.75 (1.08‐2.83)
7	1.63 (1.04‐2.56)	1.74 (1.05‐2.88)
8	1.83 (1.18‐2.83)	1.61 (0.96‐2.69)
*P* for trend	.004	.012
Trunk fat mass index (kg/m^2^)		
Per SD increase	1.15 (1.04‐1.27)	1.15 (1.04‐1.27)
Octiles^b^		
1	1.00	1.00
2	1.48 (0.99‐2.23)	1.52 (0.96‐2.41)
3	1.30 (0.84‐2.01)	1.21 (0.73‐2.01)
4	1.37 (0.88‐2.13)	1.55 (0.95‐2.53)
5	1.72 (1.12‐2.65)	1.92 (1.19‐3.11)
6	1.71 (1.10‐2.66)	1.87 (1.14‐3.06)
7	1.89 (1.21‐2.95)	2.10 (1.26‐3.48)
8	1.84 (1.18‐2.89)	1.76 (1.04‐2.98)
*P* for trend	<.001	.018

a Adjusted for age at enrollment, education, race/ethnicity, family history of breast cancer, age at menarche, age at first full‐term birth, parity, age at menopause, oral contraceptive use, hormone therapy use, physical activity, alcohol intake, smoking, and study component

b Cut‐points‐ whole body fat mass (kg):‐ ≤20.39, 27.40‐24.32, 24.33‐27.72, 27.73‐30.90, 30.91‐34.53, 34.54‐38.90,38.91‐45.93, >45.93; whole body percent fat (kg): ≤35.5, 35.6‐39.3, 39.4‐42.0, 42.1‐44.4, 44.5‐46.6, 46.7‐49.0, 49.1‐52.0, >52, trunk fat mass (kg): ≤8.23, 8.24‐10.65, 10.66‐12.62, 12.63‐14.43, 14.44‐16.44, 16.45‐18.85, 18.86‐22.33,>22.33; fat mass of right leg (kg): <3.98, 3.99‐4.66, 4.67‐5.23, 5.24‐5.81, 5.82‐6.46, 6.47‐7.24, 7.25‐8.57, >8.57; fat mass of left leg (kg): <3.87, 3.88‐4.54, 4.55‐5.09, 5.10‐5.67, 5.68‐6.31, 6.32‐7.08, 7.08‐8.39, >8.39, ratio of trunk fat mass to average of R and L leg fat mass: ≤1.66, 1.66‐1.98, 1.99‐2.21, 2.22‐2.43, 2.43‐2.68, 2.68‐2.96, 2.97‐3.39, >3.39; body fat index:‐ ≤7.85, 7.85‐9.34, 9.35‐10.64, 10.65‐11.87, 11.88‐13.29, 13.30‐14.91, 14.92‐17.54, >17.54; trunk fat mass index: ≤3.14, 3.15‐4.09, 4.10‐4.85, 4.86‐ 5.57, 5.58‐6.31, 6.32‐7.20, 7.20‐8.58, >8.58 for octiles 1, 2, 3, 4, 5, 6, 7 and 8, respectively

The associations of the DXA measures with risk of breast cancer (overall and ER+) were attenuated, but remained statistically significant after additional adjustment for lean body mass (Tables S4). Furthermore, women with a relatively high whole body fat to lean mass ratio were observed to have increased risk of breast cancer (overall and ER+; Table S5). All body fat measures were also associated with risk of breast cancer in analyses restricted to women from the OS group (Tables 4, Tables S3 and S6).

In analyses where we assessed the modifying effect of HT use, the DXA‐derived body fat measures were associated with increased risk of invasive breast cancer among never‐users (Table 3). Among current HT users, trunk fat mass, ratio of trunk fat mass to the average of right and left leg fat mass and the body fat indices were also positively associated with risk. There was also some evidence that the association of fat mass of the right leg was modified by HT use (*P*_heterogeneity_: .031) (Table S7).

**Figure 1:**
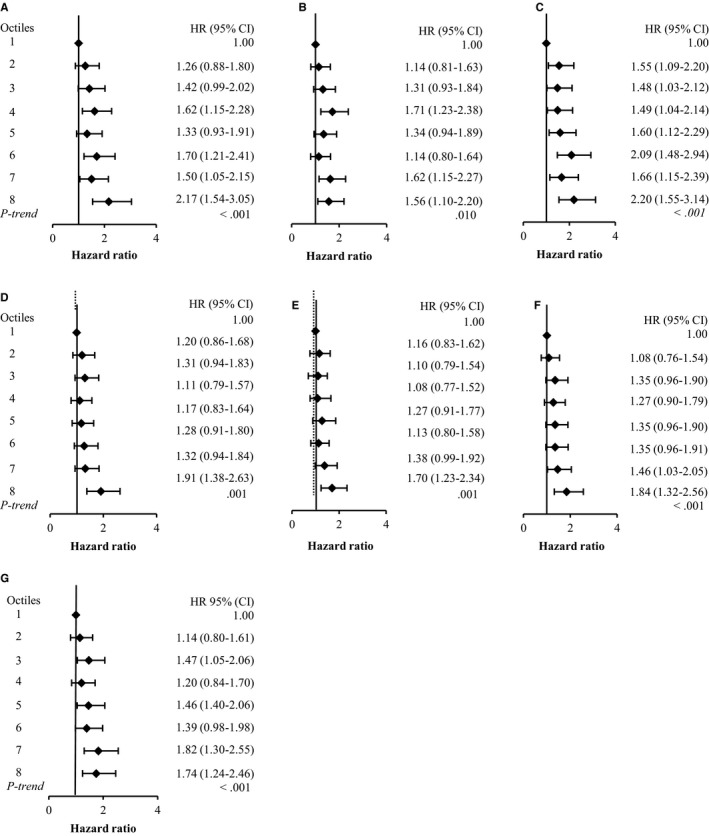
Hazard ratios and 95% CI from time‐dependent analysis for the association between DXA‐derived body fat measures and incident, invasive breast cancer in postmenopausal women. A) Whole body fat (kg); B) Whole body fat%; C) Trunk fat mass (kg); D) Fat mass of the right leg (kg); E) Fat mass of the left leg (kg); F) Fat mass index (kg/m^2^); G) Trunk fat mass index (kg/m^2^).

**Figure 2:**
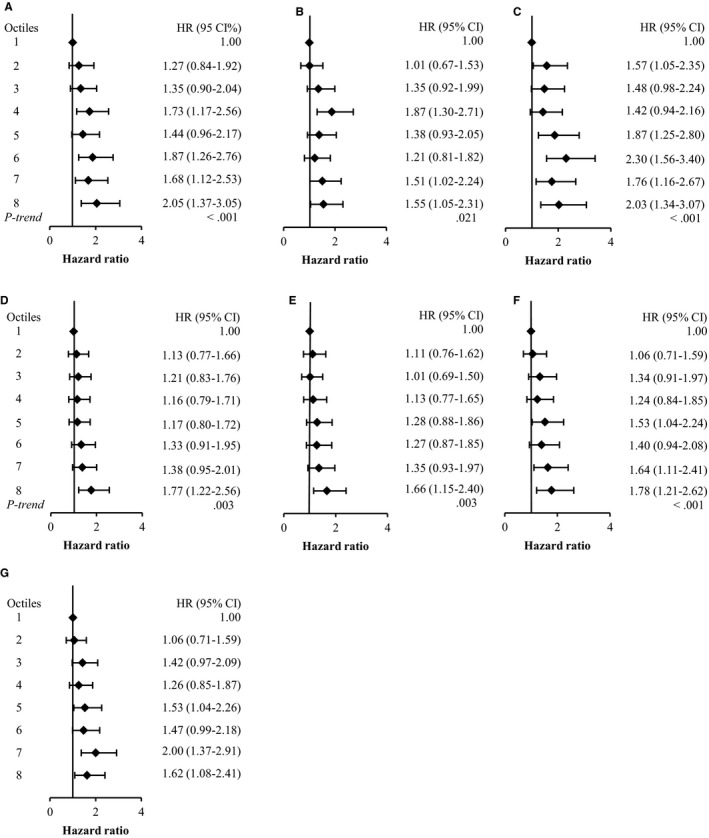
Hazard ratios and 95% CI from time‐dependent analysis for the association between DXA‐derived body fat measures and incident, invasive ER‐positive breast cancer in postmenopausal women. A) Whole body fat (kg); B) Whole body fat%; C) Trunk fat mass (kg); D) Fat mass of the right leg (kg); E) Fat mass of the left leg (kg); F) Fat mass index (kg/m^2^); G) Trunk fat mass index (kg/m^2^)

The results of our time‐dependent covariate analyses also demonstrated positive associations between all DXA‐derived body fat measures and risk of invasive breast cancer (overall and ER+; Figures 1 and 2). In particular, the highest octiles of whole body fat mass (HR: 2.17; 1.54‐3.05, 2.05; 1.37‐3.05 for overall and ER+, respectively) and trunk fat mass (HR: 2.20; 1.55‐3.14, 2.03; 1.34‐3.07 for overall and ER+, respectively) were associated with over a twofold increased risk of invasive breast cancer. In these analyses, BMI and WC were also positively associated with risk of breast cancer (overall and ER+), but, WHR was only associated overall risk of invasive breast cancer (Table S8).

There were no substantial changes in the HRs after exclusion of women with a diagnosis within two years of enrollment (Tables S9).

## 4 DISCUSSION

The results of this study confirm and extend our previous findings of positive associations between DXA‐derived and anthropometric body fat measures and risk of breast cancer (overall and ER+) in postmenopausal women.^18^ Notably, the associations for whole body fat mass and trunk fat mass with risk of breast cancer (overall and ER+) persisted after additional adjustment for the anthropometric indices (BMI, WC, or WHR). The associations between most of the DXA‐derived measures and risk of invasive breast cancer were somewhat stronger among non‐HT users than current HT users. The positive association between the baseline body fat measures and risk of invasive breast cancer were substantiated in our time‐dependent analyses.

In studies examining the associations between adiposity and risk of chronic disease, less robust methods than DXA, such as bioelectrical impedance analysis (BIA), have been widely used to determine levels of adiposity.^27^, ^28^ Compared to DXA, BIA is less sensitive in estimating % body fat, whole body fat mass and lean body mass among lean or obese individuals.^27^, ^28^ Despite its limitations, in agreement with the current study, recent findings from a large prospective study demonstrated strong positive associations between BIA‐derived body fat measures, including whole body fat mass, trunk fat mass, and body fat percent, with overall risk of breast cancer.^29^ Similar positive associations were reported in other studies that investigated the associations between BIA‐derived whole body fat mass and body fat percent and risk of breast cancer, including ER+ breast cancer.^30^, ^31^, ^32^

Anthropometric measures, particularly BMI, are also widely used as measures of adiposity.^9^, ^17^, ^33^ The utility of BMI in research settings remains questionable as it has several limitations, such as an inability to differentiate between lean and fat mass,^13^, ^15^ that may limit its ability to reliably predict the risk of diseases among persons with excess adiposity. Nevertheless, BMI has been consistently shown to have a relatively strong positive association with risk of postmenopausal invasive breast cancer.^4^, ^8^, ^10^, ^11^, ^12^ Moreover in the current study, the HRs per SD increase in BMI were of similar magnitude to those of the DXA‐derived measures, suggesting that DXA‐derived measures are not necessarily better predictors of breast cancer risk. Notwithstanding this, we observed that the associations of whole body fat mass and measures of central adiposity, including trunk fat mass, the ratio of trunk fat mass to the average of left and right leg fat, and TFMI, with risk of breast cancer (overall or ER+) were independent of BMI and other conventional breast cancer risk factors. These findings imply that DXA‐derived measures contribute to risk of postmenopausal breast cancer beyond that associated with BMI and other risk factors.^34^

Body fat composition varies with height. However, epidemiological studies have principally evaluated the associations of absolute values or percentages of the DXA‐derived measures, which do not reflect height differences, with risk of breast cancer.^23^, ^35^ Thus, such measures may not sufficiently explain potential differences in disease risk that may be apparent among individuals with similar body composition, but different heights. To compensate for this limitation, we used height‐normalized values (namely FMI and TFMI) which were proposed by VanItallie et al^23^ In the current study, both indices were associated with more than a doubling of the risk of invasive breast cancer. We are only aware of one study which examined the association of FMI with risk of breast cancer and, in this study, which was restricted to black women, no association was seen.^36^ This may be partly explained by ethnic differences in the association between body fat and risk of breast cancer. Ethnic differences in the associations of the body fat measures with risk of breast cancer were not examined in this study because the number of non‐Caucasian women with breast cancer was too small.

Findings from previous studies suggest that HT use modifies the association between adiposity and risk of invasive breast cancer.^37^, ^38^, ^39^ These studies found that the positive association between BMI and risk of breast cancer was stronger among women who had never used HT.^37^, ^38^ The weaker associations among current HT users may be partly explained by improved insulin sensitivity from chronic use of therapies with low estrogen doses; thereby, mitigating the adverse effects of insulin on breast carcinogenesis.^40^ However, there was little evidence in the present study to suggest that a woman's HT status modifies the associations between the DXA measures and risk of breast cancer.

Our findings are in accord with biological evidence which suggests that excess adiposity contributes to altered metabolism. Specifically, in an obesogenic environment, adipocytes become enlarged.^41^ These large adipocytes, which can be found in both breast adipose and visceral adipose tissue (VAT), are insulin‐resistant and hyperlipolytic, and may therefore contribute to the elevated levels of insulin, glucose, and triglycerides,^41^, ^42^, ^43^ as seen among women in the current study who had excess central adiposity. Chronic hyperglycemia is believed to stimulate tumor cell growth by causing epigenetic modifications.^1^ Moreover hyperinsulinemia can induce tumor cell proliferation and migration, at least in part by activating the PI3kinase pathway.^1^ The insulin resistance that occurs in association with excess body fat leads to downregulation of SHBG which results, in turn, in elevated circulating levels of free estrogens that contribute to the development of ER+ breast cancer.^1^ Adipocyte hypertrophy also enhances the production of pro‐inflammatory chemokines and cytokines, such as tumor necrosis factor‐α, interleukins, and leptin in human white adipose tissue,^2^, ^43^ which may stimulate mutagenesis, tumor cell proliferation, survival, migration, angiogenesis, and stem cell survival.^44^, ^45^ Moreover, a variety of molecules that are altered in association with excess body fat including the bioactive lipid prostaglandin E_2_ (PGE_2_) and leptin induce aromatase, the rate‐limiting enzyme for estrogen biosynthesis.^2^, ^43^ Thus, systemic alterations, in combination with enhanced local estrogen production, are likely to contribute to the development of hormone receptor‐positive breast cancer.^2^, ^43^

Lean body mass is thought to exert anti‐carcinogenic benefits by inhibiting cancer‐associated processes such as insulin resistance, chronic inflammation, and dysregulated lipid metabolism.^46^ In keeping with this, we observed that, after adjustment for lean body mass, the associations between the body fat measures and risk of invasive breast cancer were attenuated. Notably, in line with a previous study,^47^ our study demonstrated that women with higher body fat to lean body mass ratio had greater risk of breast cancer. This finding suggests that any potential benefit of lean body mass on risk of breast cancer may be offset by the deleterious effect of excess adiposity.

A strength of this study was the availability of body fat measures, which were obtained using DXA. DXA is a quick, non‐invasive, and highly reproducible method for directly measuring body composition. This method, however, lacks the ability to distinguish between subcutaneous and visceral abdominal adipose tissue. However, studies have shown that DXA is almost as accurate as MRI and CT in determining skeletal muscle mass and fat mass compartments.^48^ Furthermore, this is the first known study to assess the association of TFMI with risk of breast cancer. Our study also had repeated body fat measures which allowed us to assess the association of longitudinal changes in these measures with risk of breast cancer. However, a limitation of the study was that some of the variables were self‐reported. Hence, these variables may have been misclassified due to measurement error. Additionally, multiple testing was performed so that the results, while strong, need to be interpreted cautiously.

## 5 CONCLUSION

Our findings suggest that overall and central adiposity may influence the risk of postmenopausal breast cancer (overall and ER+) beyond the contributions of anthropometric measures and standard breast cancer risk factors. As this is the first known study to explore the associations between TFMI with risk of breast cancer, further studies are needed to confirm the utility of height‐normalized values for predicting the risk of breast cancer among postmenopausal women. Our findings lend support for the development of behavioral or pharmacological interventions aimed at promoting weight loss among women at high risk for breast cancer due to excess body fat.

## CONFLICT OF INTERESTS

None declared.

## WOMEN'S HEALTH INITIATIVE INVESTIGATORS

**Program Office**: (National Heart, Lung, and Blood Institute, Bethesda, MD) Jacques Roscoe, Shari Ludlum, Dale Burden, Joan McGowan, Leslie Ford, and Nancy Geller.

**Clinical Coordinating Center:** (Fred Hutchinson Cancer Research Center, Seattle, WA) Garnet Anderson, Ross Prentice, Andrea LaCroix, and Charles Kopperberg).

**Investigators and Academic Centers**: (Brigham and Women's Hospital, Harvard Medical School, Boston, MA) JoAnn E, Manson; (MedStar Health Research Institute/Howard University, Washington, DC) Barbara V Howard; (Stanford Prevention Research Center, Stanford, CA) Marcia L. Stefanick; (The Ohio State University, Columbus, OH) Rebecca Jackson; (University of Arizona, Tucson/Phoenix, AZ) Cynthia A. Thomson; (University at Buffalo, Buffalo, NY) Jean Wactawski‐Wende; (University of Florida, Gainesville/Jacksonville, FL) Marian Limacher; (University of Iowa, Iowa City/Davenport, IA) Robert Wallace; (University of Pittsburgh, Pittsburgh, PA) Lewis Kuller; (City of Hope Comprehensive Cancer Center, Duarte, CA) Rowan T. Chlebowski; (Wake Forest University School of Medicine, Winston‐Salem, NC) Sally Shumaker.

**Wome** **n's** **Health Initiative Memory Study**: (Wake Forest University School of Medicine, Winston Salem, NC) Sally Shumaker.

**Additional information**: A full list of all the investigators who have contributed to Women's Health Initiative science appears at: https://www.whi.org/researchers/Documents%2520%2520Write%2520a%2520Paper/WHI%2520Inv estigator%20Long%20List.p

## References

[1] Park J , Morley TS , Kim M , Clegg DJ , Scherer PE . Obesity and cancer‐mechanisms underlying tumour progression and recurrence. Nat Rev Endocrinol. 2014;10:455‐465. 24935119 10.1038/nrendo.2014.94 PMC4374431

[2] Khandekar MJ , Cohen P , Spiegelman BM . Molecular mechanisms of cancer development in obesity. Nat Rev Cancer. 2011;11:886‐895. 22113164 10.1038/nrc3174

[3] Kabat GC , Xue X , Kamensky V , et al. Risk of breast, endometrial, colorectal, and renal cancers in postmenopausal women in association with a body shape index and other anthropometric measures. Cancer Causes Control. 2015;26:219‐229. 25430815 10.1007/s10552-014-0501-4

[4] White AJ , Nichols HB , Bradshaw PT , Sandler DP . Overall and central adiposity and breast cancer risk in the sister study. Cancer. 2015;121:3700‐3708. 26193782 10.1002/cncr.29552 PMC4592412

[5] Pacholczak R , Klimek‐Piotrowska W , Kuszmiersz P . Associations of anthropometric measures on breast cancer risk in pre‐ and postmenopausal women‐a case‐control study. J Physiol Anthropol. 2016;35:7. 26951106 10.1186/s40101-016-0090-x PMC4782382

[6] Phipps AI , Chlebowski RT , Prentice R , et al. Body size, physical activity, and risk of triple‐negative and estrogen receptor‐positive breast cancer. Cancer Epidemiol Biomarkers Prev. 2011;20:454‐463. 21364029 10.1158/1055-9965.EPI-10-0974 PMC3064558

[7] Pinheiro RL , Sarian LO , Pinto‐Neto AM , Morais S , Costa‐Paiva L . Waist circumference and waist to hip ratio do not contribute additional information on hormone receptor status of breast tumors in obese women. Breast J. 2010;16:323‐324. 20408825 10.1111/j.1524-4741.2010.00902.x

[8] Harding JL , Shaw JE , Anstey KJ , et al. Comparison of anthropometric measures as predictors of cancer incidence: a pooled collaborative analysis of 11 Australian cohorts. Int J Cancer. 2015;137:1699‐1708. 25810218 10.1002/ijc.29529

[9] Okorodudu DO , Jumean MF , Montori VM , et al. Diagnostic performance of body mass index to identify obesity as defined by body adiposity: a systematic review and meta‐analysis. Int J Obes. 2010;34:791‐799. 10.1038/ijo.2010.5 20125098

[10] Chen G‐C , Chen S‐J , Zhang R , et al. Central obesity and risks of pre‐ and postmenopausal breast cancer: a dose–response meta‐analysis of prospective studies. Obes Rev. 2016;17:1167‐1177. 27432212 10.1111/obr.12443

[11] Gaudet MM , Carter BD , Patel AV , Teras LR , Jacobs EJ , Gapstur SM . Waist circumference, body mass index, and postmenopausal breast cancer incidence in the Cancer Prevention Study‐II Nutrition Cohort. Cancer Causes Control. 2014;25:737‐745. 24715420 10.1007/s10552-014-0376-4

[12] Tamaki K , Tamaki N , Terukina S , et al. The correlation between body mass index and breast cancer risk or estrogen receptor status in Okinawan women. Tohoku J Exp Med. 2014;234:169‐174. 25283589 10.1620/tjem.234.169

[13] Alemán JO , Iyengar NM , Walker JM , et al. Effects of rapid weight loss on systemic and adipose tissue inflammation and metabolism in obese postmenopausal women. J Endocr Soc. 2017;1:625‐637. 29264516 10.1210/js.2017-00020 PMC5686624

[14] Canoy D , Boekholdt SM , Wareham N , et al. Body fat distribution and risk of coronary heart disease in men and women in the european prospective investigation into cancer and nutrition in norfolk cohort. Circulation. 2007;116:2933‐2943. 18071080 10.1161/CIRCULATIONAHA.106.673756

[15] Blew RM , Sardinha LB , Milliken LA , et al. Assessing the validity of body mass index standards in early postmenopausal women. Obes Res. 2002;10:799‐808. 12181389 10.1038/oby.2002.108

[16] Iyengar NM , Brown KA , Zhou XK , et al. Metabolic obesity, adipose inflammation and elevated breast aromatase in women with normal body mass index. Cancer Prev Res (Phila). 2017;10:235‐243. 28270386 10.1158/1940-6207.CAPR-16-0314 PMC5380584

[17] Goodpaster BH . Measuring body fat distribution and content in humans. Curr Opin Clin Nutr Metab Care. 2002;5:481‐487. 12172470 10.1097/00075197-200209000-00005

[18] Rohan TE , Heo M , Choi L , et al. Body fat and breast cancer risk in postmenopausal women: a longitudinal study. J Cancer Epidemiol. 2013;2013:754815. 23690776 10.1155/2013/754815 PMC3649193

[19] Iyengar NM , Arthur R , Manson JE , et al. Association of body fat and risk of breast cancer in postmenopausal women with normal body mass index: a secondary analysis of a randomized clinical trial and observational study. JAMA Oncol. 2019;5:155‐163. 30520976 10.1001/jamaoncol.2018.5327 PMC6439554

[20] The Women's Health Initiative Study Group . Design of the women's health initiative clinical trial and observational study. Control Clin Trials. 1998;19:61‐109. 9492970 10.1016/s0197-2456(97)00078-0

[21] Carty CL , Kooperberg C , Neuhouser ML , et al. Low‐fat dietary pattern and change in body‐composition traits in the Women's Health Initiative Dietary Modification Trial. Am J Clin Nutr. 2011;93:516‐524. 21177798 10.3945/ajcn.110.006395 PMC3041598

[22] Chen Z , Bassford T , Green SB , et al. Postmenopausal hormone therapy and body composition—a substudy of the estrogen plus progestin trial of the Women's Health Initiative. Am J Clin Nutr. 2005;82:651‐656. 16155280 10.1093/ajcn.82.3.651

[23] VanItallie TB , Yang MU , Heymsfield SB , Funk RC , Boileau RA . Height‐normalized indices of the body's fat‐free mass and fat mass: potentially useful indicators of nutritional status. Am J Clin Nutr. 1990;52:953‐959. 2239792 10.1093/ajcn/52.6.953

[24] Gavi S , Feiner JJ , Melendez MM , Mynarcik DC , Gelato MC , McNurlan MA . Limb fat to trunk fat ratio in elderly persons is a strong determinant of insulin resistance and adiponectin levels. J Gerontol A Biol Sci Med Sci. 2007;62:997‐1001. 17895438 10.1093/gerona/62.9.997

[25] Curb JDavid , Mctiernan A , Heckbert SR , et al. Outcomes ascertainment and adjudication methods in the Women's Health Initiative. Ann Epidemiol. 2003;13:S122‐S128. 14575944 10.1016/s1047-2797(03)00048-6

[26] Orsini N , Greenland S . A procedure to tabulate and plot results after flexible modeling of a quantitative covariate. Stata J. 2011;11:1‐29.

[27] Faria SL , Faria OP , Cardeal MDA , Ito MK . Validation study of multi‐frequency bioelectrical impedance with dual‐energy X‐ray absorptiometry among obese patients. Obesity Surg. 2014;24:1476‐1480. 10.1007/s11695-014-1190-5 24464546

[28] Sun G , French CR , Martin GR , et al. Comparison of multifrequency bioelectrical impedance analysis with dual‐energy X‐ray absorptiometry for assessment of percentage body fat in a large, healthy population. Am J Clin Nutr. 2005;81:74‐78. 15640463 10.1093/ajcn/81.1.74

[29] Wenji G , Key TJ , Reeves GK . Adiposity and breast cancer risk in postmenopausal women: results from the UK Biobank prospective cohort. Int J Cancer. 2018;143:1037‐1046. 29569713 10.1002/ijc.31394 PMC6099222

[30] Lahmann PH , Lissner L , Gullberg BO , Olsson H , Berglund G . A prospective study of adiposity and postmenopausal breast cancer risk: the Malmö diet and cancer study. Int J Cancer. 2003;103:246‐252. 12455040 10.1002/ijc.10799

[31] Krebs EE , Taylor BC , Cauley JA , Stone KL , Bowman PJ , Ensrud KE . Measures of adiposity and risk of breast cancer in older postmenopausal women. J Am Geriatr Soc. 2006;54:63‐69. 16420199 10.1111/j.1532-5415.2005.00541.x

[32] MacInnis RJ , English DR , Gertig DM , Hopper JL , Giles GG . Body size and composition and risk of postmenopausal breast cancer. Cancer Epidemiol Biomarkers Prev. 2004;13:2117. 15598769

[33] Duren DL , Sherwood RJ , Czerwinski SA , et al. Body composition methods: comparisons and interpretation. J Diabetes Sci Technol. 2008;2:1139‐1146. 19885303 10.1177/193229680800200623 PMC2769821

[34] Renehan AG , Zwahlen M , Egger M . Adiposity and cancer risk: new mechanistic insights from epidemiology. Nat Rev Cancer. 2015;15:484‐498. 26205341 10.1038/nrc3967

[35] Kyle UG , Schutz Y , Dupertuis YM , Pichard C . Body composition interpretation: contributions of the fat‐free mass index and the body fat mass index. Nutrition. 2003;19:597‐604. 12831945 10.1016/s0899-9007(03)00061-3

[36] Bandera EV , Chandran U , Zirpoli G , et al. Body fatness and breast cancer risk in women of African ancestry. BMC Cancer. 2013;13:475. 24118876 10.1186/1471-2407-13-475 PMC3853021

[37] Hou N , Hong S , Wang W , Olopade OI , Dignam JJ , Huo D . Hormone replacement therapy and breast cancer: heterogeneous risks by race, weight, and breast density. JNCI. 2013;105:1365‐1372. 24003037 10.1093/jnci/djt207 PMC3776262

[38] Li CI , Malone KE , Daling JR . Interactions between body mass index and hormone therapy and postmenopausal breast cancer risk (United States). Cancer Causes Control. 2006;17(5):695‐703. 16633917 10.1007/s10552-005-0001-7

[39] Morimoto LM , White E , Chen Z , et al. Obesity, body size, and risk of postmenopausal breast cancer: the women's health initiative (United States). Cancer Causes Control. 2002;13:741‐751. 12420953 10.1023/a:1020239211145

[40] Cui Y , Deming‐Halverson SL , Beeghly‐Fadiel A , et al. Interactions of hormone replacement therapy, body weight, and bilateral oophorectomy in breast cancer risk. Clin Cancer Res. 2014;20:1169. 24423614 10.1158/1078-0432.CCR-13-2094 PMC3972811

[41] Mohsen IM . Subcutaneous and visceral adipose tissue: structural and functional differences. Obes Rev. 2010;11:11‐18. 19656312 10.1111/j.1467-789X.2009.00623.x

[42] Morris PG , Hudis CA , Giri D , et al. Inflammation and increased aromatase expression occur in the breast tissue of obese women with breast cancer. Cancer Prev Res. 2011;4:1021‐1029. 10.1158/1940-6207.CAPR-11-0110 PMC3131426 21622727

[43] Brown KA , Iyengar NM , Zhou XK , et al. Menopause is a determinant of breast aromatase expression and its associations with BMI, inflammation, and systemic markers. J Clin Endocrinol Metab. 2017;102:1692‐1701. 28323914 10.1210/jc.2016-3606 PMC5443335

[44] Ramos‐Nino ME . The role of chronic inflammation in obesity‐associated cancers. ISRN Oncol. 2013;2013:697521. 23819063 10.1155/2013/697521 PMC3683483

[45] Reuter S , Gupta SC , Chaturvedi MM , Aggarwal BB . Oxidative stress, inflammation, and cancer: how are they linked? Free Radic Biol Med. 2010;49:1603‐1616. 20840865 10.1016/j.freeradbiomed.2010.09.006 PMC2990475

[46] McDonald C , Bauer J , Capra S . Body composition and breast cancer – the role of lean body mass. 2011; Available at: https://cancerforum.org.au/forum/2011/july/body-composition-and-breast-cancer-the-role-of-lean-body-mass/. Accessed February 26, 2019.

[47] Ronco AL , Boeing H , De Stefani E , Schulz M , Schulze M , Pischon T . A case‐control study on fat‐to‐muscle ratio and risk of breast cancer. Nutr Cancer. 2009;61:466‐474. 19838918 10.1080/01635580902725995

[48] Guglielmi G , Ponti F , Agostini M , Amadori M , Battista G , Bazzocchi A . The role of DXA in sarcopenia. Aging Clin Exp Res. 2016;28:1047‐1060. 27256078 10.1007/s40520-016-0589-3

