# Supplementary material for: The association between DXA‐derived body fat measures and breast cancer risk among postmenopausal women in the Women's Health Initiative
Source: Cancer Med. 2019 Dec 25;9(4):1581–99. doi: 10.1002/cam4.2690 (PMC7013066; doi:10.1002/cam4.2690)
Supplement: Supplementary file 1 [file CAM4-9-1581-s001.docx]

Supplementary material

Total number of women recruited in the Women’s Health Initiative cohort

**N = 161,808**

**Excluded**

- 108 women who were missing baseline DXA measures
- 325 women with a previous history of breast cancer
- 29 women with missing information on time to follow-up

Number of women included in analyses

**N=10,931**

Number of women with available DXA measurements

**N= 11,393**

**Figure S1: Schematic diagram of study population**


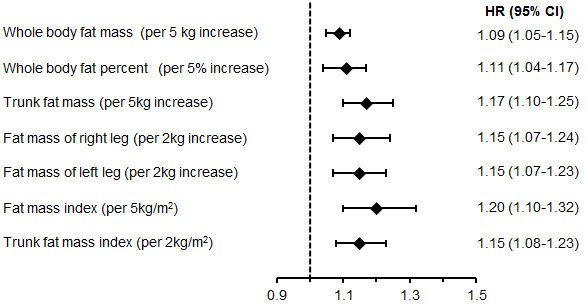


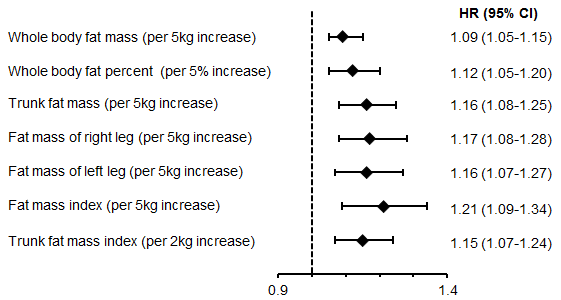


**A**

**B**

**Figure S2: HR and 95% CI for the associations of DXA-derived body fat measures with risk of invasive breast cancer. A) Overall; B) ER-positive**

**D**

**A**

**E**

**C**

**B**

**Figure S3: Multivariable HRs (solid line) with 95% CI (dashed lines) for the association of body fat measures with risk of ER+ breast cancer among postmenopausal women from the Women’s Health Initiative cohort. Models were adjusted for age at enrollment, education, race, family history of breast cancer, age at menarche, age at first full-term birth, parity, age at menopause, oral contraceptive use, hormone therapy use, physical activity, alcohol intake, smoking and study component. Median values of the body fat measures of the first quartile were used as the reference (21kg, 36%, 9kg, 8kg/m^2^ and 3kg/m^2^ for whole body fat mass, whole body fat percent, trunk fat mass, fat mass index and trunk fat mass index, respectively). P-values for non-linearity (reading from A to E) were 0.014, 0.062, 0.024, 0.012, and 0.015, respectively.**

**Table S1: Hazard ratios and 95% CI for the association between baseline anthropometric measures and incident, invasive breast cancer in postmenopausal women**

|  | Cases/person-years | Age-adjusted HR (95% CI)^a^ | Multivariable-adjusted HR (95% CI)^b^ | Multivariable-adjusted HR (95% CI)^c^ | Multivariable-adjusted HR (95% CI)^d^ |
| --- | --- | --- | --- | --- | --- |
| BMI (kg/m^2^) |  |  |  |  |  |
| Per SD increase |  | 1.16 (1.08-1.25) | 1.19 (1.10-1.28) | 1.03 (0.86-1.24) | 1.02 (0.87-1.20) |
| Octiles^e^ |  |  |  |  |  |
| 1 | 63/19596.0 | 1.00 | 1.00 | 1.00 | 1.00 |
| 2 | 64/19902.5 | 1.00 (0.71-1.42) | 1.07 (0.76-1.52) | 1.03 (0.72-1.48) | 1.02 (0.71-1.45) |
| 3 | 76/20031.2 | 1.18 (0.85-1.65) | 1.23 (0.88-1.73) | 1.16 (0.81-1.66) | 1.13 (0.79-1.62) |
| 4 | 73/19617.0 | 1.16 (0.83-1.62) | 1.28 (0.91-1.80) | 1.17 (0.80-1.72) | 1.14 (0.78-1.66) |
| 5 | 99/19714.3 | 1.56 (1.14-2.14) | 1.71 (1.24-2.36) | 1.54 (1.05-2.26) | 1.48 (1.01-2.16) |
| 6 | 70/19453.9 | 1.12 (0.80-1.57) | 1.23 (0.87-1.75) | 1.08 (0.69-1.67) | 1.03 (0.67-1.58) |
| 7 | 90/18980.9 | 1.49 (1.08-2.05) | 1.64 (1.18-2.29) | 1.38 (0.85-2.22) | 1.30 (0.81-2.06) |
| 8 | 101/18408.9 | 1.74 (1.27-2.38) | 1.96 (1.42-2.72) | 1.51 (0.82-2.78) | 1.40 (0.79-2.48) |
| *P for trend* |  | <0.001 | <0.001 | 0.224 | 0.314 |
|  |  |  |  |  |  |
| Waist circumference (cm.) |  |  |  |  |  |
| Per SD increase |  | 1.18 (1.09-1.27) | 1.20 (1.11-1.30) | 1.10 (0.96-1.27) | 1.08 (0.92-1.26) |
| Octiles^e^ |  |  |  |  |  |
| 1 | 71/21703.6 | 1.00 | 1.00 | 1.00 | 1.00 |
| 2 | 72/22223.7 | 0.99 (0.71-1.37) | 1.03 (0.74-1.43) | 0.98 (0.71-1.37) | 0.97 (0.69-1.35) |
| 3 | 71/19159.9 | 1.13 (0.81-1.57) | 1.19 (0.86-1.66) | 1.09 (0.77-1.55) | 1.06 (0.75-1.51) |
| 4 | 62/17899.3 | 1.06 (0.75-1.49) | 1.13 (0.80-1.60) | 1.01 (0.70-1.46) | 0.97 (0.66-1.42) |
| 5 | 100/21620.6 | 1.41 (1.04-1.92) | 1.53 (1.12-2.09) | 1.32 (0.93-1.88) | 1.26 (0.87-1.83) |
| 6 | 84/17429.6 | 1.47 (1.07-2.02) | 1.60 (1.16-2.21) | 1.33 (0.91-1.95) | 1.26 (0.83-1.89) |
| 7 | 89/18542.6 | 1.47 (1.08-2.01) | 1.61 (1.17-2.22) | 1.27 (0.84-1.93) | 1.19 (0.76-1.87) |
| 8 | 89/17381.8 | 1.58 (1.16-2.16) | 1.73 (1.25-2.39) | 1.23 (0.75-2.04) | 1.13 (0.65-1.96) |
| *P for trend* |  | <0.001 | <0.001 | 0.088 | 0.187 |
|  |  |  |  |  |  |
| WHR |  |  |  |  |  |
| Per SD increase |  | 1.12 (1.04-1.20) | 1.13 (1.05-1.21) | 1.09 (1.01-1.17) | 1.07 (0.99-1.16) |
| Octiles^e^ |  |  |  |  |  |
| 1 | 65/21067.5 | 1.00 | 1.00 | 1.00 | 1.00 |
| 2 | 76/20414.3 | 1.20 (0.86-1.68) | 1.25 (0.89-1.74) | 1.21 (0.87-1.68) | 1.19 (0.85-1.66) |
| 3 | 81/19880.8 | 1.32 (0.95-1.83) | 1.37 (0.99-1.91) | 1.30 (0.93-1.81) | 1.27 (0.91-1.77) |
| 4 | 70/19445.0 | 1.16 (0.83-1.63) | 1.21 (0.86-1.70) | 1.13 (0.81-1.60) | 1.10 (0.78-1.55) |
| 5 | 91/19887.7 | 1.48 (1.08-2.04) | 1.58 (1.14-2.18) | 1.43 (1.03-1.98) | 1.37 (0.98-1.91) |
| 6 | 79/18936.9 | 1.35 (0.97-1.88) | 1.46 (1.05-2.04) | 1.30 (0.93-1.83) | 1.25 (0.88-1.76) |
| 7 | 91/18467.0 | 1.60 (1.16-2.20) | 1.69 (1.22-2.34) | 1.47 (1.05-2.05) | 1.39 (0.99-1.96) |
| 8 | 85/17790.5 | 1.55 (1.12-2.14) | 1.65 (1.19-2.30) | 1.42 (1.01-1.99) | 1.32 (0.92-1.87) |
| *P for trend* |  | 0.001 | <0.001 | 0.027 | 0.103 |

^a^Adjusted for age at enrollment

^b^Adjusted for age at enrollment, education, race/ethnicity, family history of breast cancer, personal history of diabetes, age at menarche, age at first full-term birth, parity, age at menopause, oral contraceptive use, hormone therapy, physical activity, alcohol intake, and study component

^c^Also adjusted for whole body fat mass

^d^Also adjusted for trunk fat mass

^e^Cutpoints- BMI (kg/m^2^): ≤22.15, 22.16-24.00, 24.01-25.60, 25.61-27.19, 27.20-29.07, 29.08-31.31, 31.32-34.87, >34.87; waist circumference (cm): ≤71, 72-76, 77-80, 81-84, 85-89, 90-94, 95-102, >102; waist to hip ratio: ≤0.723, 0.724-.751, 0.752-0.774, 0.775-0.797, 0.798-0.821, 0.822-0.849, 0.850-0.890, >0.890 for octiles 1, 2, 3, 4, 5, 6, 7 and 8, respectively

**Table S2: Hazard ratios and 95% CI for the association between baseline anthropometric measures and incident, ER-positive breast cancer in postmenopausal women**

|  | Cases/person-years | Age-adjusted HR (95% CI)^a^ | Multivariable-adjusted HR (95% CI)^b^ | Multivariable-adjusted HR (95% CI)^c^ | Multivariable-adjusted HR (95% CI)^d^ |
| --- | --- | --- | --- | --- | --- |
| BMI (kg/m^2^) |  |  |  |  |  |
| Per SD increase |  | 1.12 (1.03-1.22) | 1.18 (1.08-1.29) | 0.95 (0.76-1.19) | 0.99 (0.83-1.21) |
| Octiles^e^ |  |  |  |  |  |
| 1 | 49/19596.0 | 1.00 | 1.00 | 1.00 | 1.00 |
| 2 | 51/19902.5 | 1.02 (0.69-1.52) | 1.12 (0.76-1.66) | 1.06 (0.71-1.59) | 1.07 (0.71-1.59) |
| 3 | 60/20031.2 | 1.20 (0.82-1.75) | 1.30 (0.89-1.90) | 1.18 (0.79-1.78) | 1.19 (0.80-1.79) |
| 4 | 54/19617.0 | 1.10 (0.75-1.62) | 1.28 (0.86-1.89) | 1.12 (0.72-1.74) | 1.14 (0.74-1.76) |
| 5 | 77/19714.3 | 1.56 (1.09-2.24) | 1.80 (1.25-2.59) | 1.53 (0.98-2.38) | 1.56 (1.01-2.41) |
| 6 | 51/19453.9 | 1.05 (0.71-1.55) | 1.24 (0.83-1.85) | 1.01 (0.61-1.69) | 1.04 (0.63-1.71) |
| 7 | 72/18980.9 | 1.54 (1.07-2.21) | 1.85 (1.27-2.69) | 1.42 (0.82-2.47) | 1.47 (0.86-2.50) |
| 8 | 69/18408.9 | 1.55 (1.07-2.24) | 1.94 (1.33-2.84) | 1.31 (0.64-2.70) | 1.40 (0.72-2.72) |
| *P for trend* |  | 0.002 | <0.001 | 0.302 | 0.243 |
|  |  |  |  |  |  |
| Waist circumference (cm.) |  |  |  |  |  |
| Per SD increase |  | 1.14 (1.04-1.24) | 1.19 (1.09-1.30) | 1.04 (0.88-1.23) | 1.03 (0.85-1.24) |
| Octiles^e^ |  |  |  |  |  |
| 1 | 59/21703.6 | 1.00 | 1.00 | 1.00 | 1.00 |
| 2 | 50/22223.7 | 0.83 (0.57-1.20) | 0.87 (0.60-1.28) | 0.81 (0.55-1.19) | 0.80 (0.54-1.18) |
| 3 | 56/19159.9 | 1.07 (0.74-1.55) | 1.17 (0.81-1.69) | 1.02 (0.69-1.50) | 1.00 (0.67-1.48) |
| 4 | 52/17899.3 | 1.07 (0.74-1.55) | 1.19 (0.82-1.73) | 0.99 (0.66-1.49) | 0.96 (0.63-1.47) |
| 5 | 73/21620.6 | 1.24 (0.88-1.75) | 1.41 (0.99-1.99) | 1.12 (0.75-1.67) | 1.08 (0.71-1.65) |
| 6 | 66/17429.6 | 1.39 (0.98-1.98) | 1.61 (1.12-2.30) | 1.20 (0.78-2.86) | 1.16 (0.73-1.84) |
| 7 | 69/18542.6 | 1.38 (0.97-1.95) | 1.62 (1.14-2.32) | 1.13 (0.70-1.81) | 1.08 (0.65-1.81) |
| 8 | 58/17381.8 | 1.26 (0.87-1.80) | 1.48 (1.02-2.15) | 0.87 (0.49-1.57) | 0.83 (0.43-1.59) |
| *P for trend* |  | 0.002 | <0.001 | 0.322 | 0.366 |
|  |  |  |  |  |  |
| WHR |  |  |  |  |  |
| Per SD increase |  | 1.10 (1.02-1.19) | 1.12 (1.04-1.22) | 1.08 (0.99-1.18) | 1.16 (0.97-1.17) |
| Octiles^e^ |  |  |  |  |  |
| 1 | 54/21067.5 | 1.00 | 1.00 | 1.00 | 1.00 |
| 2 | 60/20414.3 | 1.14 (0.79-1.64) | 1.18 (0.82-1.71) | 1.14 (0.79-1.66) | 1.12 (0.78-1.63) |
| 3 | 56/19880.8 | 1.09 (0.75-1.59) | 1.16 (0.79-1.68) | 1.09 (0.74-1.58) | 1.06 (0.73-1.55) |
| 4 | 60/19444.9 | 1.19 (0.82-1.72) | 1.27 (0.88-1.84) | 1.18 (0.81-1.71) | 1.14 (0.79-1.67) |
| 5 | 67/19887.7 | 1.31 (0.91-1.87) | 1.44 (1.00-2.07) | 1.28 (0.88-1.85) | 1.23 (0.85-1.79) |
| 6 | 61/18936.9 | 1.25 (0.86-1.80) | 1.41 (0.97-2.05) | 1.24 (0.85-1.82) | 1.19 (0.81-1.75) |
| 7 | 58/18467.0 | 1.22 (0.84-1.76) | 1.34 (0.92-1.96) | 1.14 (0.78-1.68) | 1.09 (0.73-1.62) |
| 8 | 67/17790.5 | 1.46 (1.02-2.09) | 1.63 (1.13-2.36) | 1.37 (0.94-2.01) | 1.28 (0.86-1.90) |
| *P for trend* |  | 0.039 | 0.006 | 0.150 | 0.324 |

^a^Adjusted for age at enrollment

^b^Adjusted for age at enrollment, education, race/ethnicity, family history of breast cancer, personal history of diabetes, age at menarche, age at first full-term birth, parity, age at menopause, oral contraceptive use, hormone therapy, physical activity, alcohol intake, and study component

^c^Also adjusted for whole body fat mass

^d^Also adjusted for trunk fat mass

^e^Cutpoints- BMI (kg/m^2^): ≤22.15, 22.16-24.00, 24.01-25.60, 25.61-27.19, 27.20-29.07, 29.08-31.31, 31.32-34.87, >34.87; waist circumference (cm): ≤71, 72-76, 77-80, 81-84, 85-89, 90-94, 95-102, >102; waist to hip ratio: ≤0.723, 0.724-.751, 0.752-0.774, 0.775-0.797, 0.798-0.821, 0.822-0.849, 0.850-0.890, >0.890 for octiles 1, 2, 3, 4, 5, 6, 7 and 8, respectively

**Table S3: Hazard ratios and 95% CI for the association between baseline anthropometric measures and incident, invasive breast cancer in postmenopausal women from the Observational Study group**

|  | Overall | ER-positive |
| --- | --- | --- |
|  | HR (95% CI) | |
| BMI (kg/m^2^) |  |  |
| Per SD increase | 1.14 (1.03-1.26) | 1.12 (0.99-1.26) |
| Octiles^a^ |  |  |
| 1 | 1.00 | 1.00 |
| 2 | 1.06 (0.69-1.62) | 1.01 (0.63-1.62) |
| 3 | 1.30 (0.86-1.96) | 1.28 (0.81-2.02) |
| 4 | 1.29 (0.84-1.99) | 1.28 (0.80-2.06) |
| 5 | 1.86 (1.25-2.76) | 1.73 (1.11-2.71) |
| 6 | 1.15 (0.72-1.81) | 1.15 (0.69-1.91) |
| 7 | 1.68 (1.08-2.60) | 1.76 (1.08-2.86) |
| 8 | 1.68 (1.08-2.59) | 1.51 (0.92-2.50) |
| *P for trend* | 0.021 | 0.021 |
|  |  |  |
| Waist circumference (cm.) |  |  |
| Per SD increase | 1.17 (1.06-1.30) | 1.15 (1.02-1.30) |
| Octiles^a^ |  |  |
| 1 | 1.00 | 1.00 |
| 2 | 1.05 (0.70-1.57) | 0.92 (0.58-1.43) |
| 3 | 1.09 (0.71-1.65) | 0.98 (0.61-1.57) |
| 4 | 1.24 (0.82-1.91) | 1.23 (0.78-1.96) |
| 5 | 1.36 (0.91-2.04) | 1.15 (0.73-1.83) |
| 6 | 1.67 (1.11-2.51) | 1.82 (1.17-2.82) |
| 7 | 1.52 (0.99-2.34) | 1.43 (0.88-2.32) |
| 8 | 1.51 (0.98-2.32) | 1.22 (0.76-2.02) |
| *P for trend* | 0.005 | 0.031 |
|  |  |  |
| WHR |  |  |
| Per SD increase | 1.10 (1.00-1.20) | 1.08 (0.97-1.20) |
| Octiles^a^ |  |  |
| 1 | 1.00 | 1.00 |
| 2 | 1.51 (0.99-2.29) | 1.52 (0.96-2.39) |
| 3 | 1.52 (0.99-2.32) | 1.35 (0.83-2.18) |
| 4 | 1.30 (0.83-2.04) | 1.34 (0.82-2.18) |
| 5 | 1.72 (1.12-2.64) | 1.43 (0.88-2.34) |
| 6 | 1.49 (0.95-2.34) | 1.52 (0.93-2.49) |
| 7 | 1.51 (0.96-2.37) | 1.09 (0.64-1.88) |
| 8 | 1.74 (1.12-2.71) | 1.70 (1.04-2.79) |
| *P for trend* | 0.042 | 0.207 |

Adjusted for age at enrollment, education, race/ethnicity, family history of breast cancer, personal history of diabetes, age at menarche, age at first full-term birth, parity, age at menopause, oral contraceptive use, hormone therapy, physical activity, alcohol intake, and study component

^a^Cutpoints- BMI (kg/m^2^): ≤22.15, 22.16-24.00, 24.01-25.60, 25.61-27.19, 27.20-29.07, 29.08-31.31, 31.32-34.87, >34.87; waist circumference (cm): ≤71, 72-76, 77-80, 81-84, 85-89, 90-94, 95-102, >102; waist to hip ratio: ≤0.723, 0.724-.751, 0.752-0.774, 0.775-0.797, 0.798-0.821, 0.822-0.849, 0.850-0.890, >0.890 for octiles 1, 2, 3, 4, 5, 6, 7 and 8, respectively

**Table S4: Hazard ratios and 95% CI for the associations of baseline DXA-derived body fat measures and incident, invasive breast cancer in postmenopausal women after additional adjustment for lean body mass**

|  | Overall | ER-positive |
| --- | --- | --- |
|  | HR (95% CI) | |
| **Whole body fat mass (kg)** |  |  |
| Per SD increase | 1.10 (0.99-1.21) | 1.14 (1.02-1.28) |
| Octiles^a^ |  |  |
| 1 | 1.00 | 1.00 |
| 2 | 1.17 (0.82-1.67) | 1.10 (0.73-1.65) |
| 3 | 1.36 (0.96-1.92) | 1.27 (0.85-1.88) |
| 4 | 1.28 (0.90-1.83) | 1.32 (0.89-1.96) |
| 5 | 1.67 (1.19-2.34) | 1.76 (1.21-2.57) |
| 6 | 1.60 (1.13-2.26) | 1.56 (1.05-2.32) |
| 7 | 1.42 (1.09-2.24) | 1.53 (1.01-2.31) |
| 8 | 1.64 (1.11-2.41) | 1.68 (1.08-2.61) |
| *P for trend* | 0.009 | 0.008 |
| **Whole body fat percent** |  |  |
| Per SD increase | 1.12 (1.02-1.22) | 1.15 (1.04-1.27) |
| Octiles^a^ |  |  |
| 1 | 1.00 | 1.00 |
| 2 | 1.13 (0.80-1.60) | 1.15 (0.77-1.71) |
| 3 | 1.43 (1.03-1.99) | 1.50 (1.03-2.19) |
| 4 | 1.40 (1.00-1.96) | 1.42 (0.96-2.09) |
| 5 | 1.50 (1.07-2.09) | 1.63 (1.12-2.39) |
| 6 | 1.41 (1.00-1.98) | 1.51 (1.02-2.24) |
| 7 | 1.69 (1.21-2.35) | 1.61 (1.09-2.38) |
| 8 | 1.39 (0.98-1.98) | 1.61 (1.08-2.39) |
| *P for trend* | 0.002 | 0.008 |
| **Trunk fat mass (kg)** |  |  |
| Per SD increase | 1.11 (1.01-1.23) | 1.14 (1.02-1.27) |
| Octiles^a^ |  |  |
| 1 | 1.00 | 1.00 |
| 2 | 1.62 (1.13-2.30) | 1.62 (1.08-2.42) |
| 3 | 1.65 (1.16-2.36) | 1.68 (1.12-2.51) |
| 4 | 1.29 (0.88-1.87) | 1.28 (0.83-1.96) |
| 5 | 1.95 (1.38-2.77) | 2.16 (1.46-3.19) |
| 6 | 1.86 (1.30-2.67) | 1.96 (1.30-2.96) |
| 7 | 1.61 (1.10-2.35) | 1.74 (1.13-2.68) |
| 8 | 1.99 (1.35-2.93) | 1.97 (1.26-3.08) |
| *P for trend* | 0.004 | <0.007 |
| **Fat mass of right leg (kg)** |  |  |
| Per SD increase | 1.07 (0.98-1.17) | 1.12 (1.01-1.24) |
| Octiles^a^ |  |  |
| 1 | 1.00 | 1.00 |
| 2 | 1.30 (0.92-1.84) | 1.29 (0.86-1.93) |
| 3 | 1.21 (0.85-1.73) | 1.11 (0.73-1.68) |
| 4 | 1.33 (0.94-1.89) | 1.41 (0.95-2.10) |
| 5 | 1.30 (0.92-1.84) | 1.47 (0.99-2.18) |
| 6 | 1.34 (0.94-1.91) | 1.46 (0.98-2.17) |
| 7 | 1.44 (1.01-2.04) | 1.60 (1.07-2.38) |
| 8 | 1.61 (1.12-2.32) | 1.64 (1.07-2.50) |
| P for trend | 0.015 | 0.010 |
| **Fat mass of left leg (kg)** |  |  |
| Per SD increase | 1.06 (0.97-1.16) | 1.11 (0.99-1.23) |
| Octiles^a^ |  |  |
| 1 | 1.00 | 1.00 |
| 2 | 1.25 (0.89-1.77) | 1.15 (0.77-1.72) |
| 3 | 1.22 (0.86-1.73) | 1.14 (0.77-1.71) |
| 4 | 1.26 (0.89-1.77) | 1.28 (0.86-1.89) |
| 5 | 1.39 (0.99-1.95) | 1.48 (1.01-2.16) |
| 6 | 1.27 (0.90-1.80) | 1.38 (0.93-2.03) |
| 7 | 1.40 (0.99-1.98) | 1.43 (0.97-2.12) |
| 8 | 1.41 (0.98-2.04) | 1.38 (0.91-2.11) |
| P for trend | 0.078 | 0.063 |
| **Ratio of trunk fat mass to average of R and L leg fat mass** |  |  |
| Octiles^a^ |  |  |
| 1 | 1.00 | 1.00 |
| 2 | 1.35 (0.96-1.90) | 1.28 (0.86-1.89) |
| 3 | 1.68 (1.21-2.34) | 1.86 (1.29-2.69) |
| 4 | 1.55 (1.11-2.17) | 1.63 (1.12-2.39) |
| 5 | 1.31 (0.92-1.85) | 1.23 (0.82-1.85) |
| 6 | 1.47 (1.04-2.07) | 1.56 (1.06-2.31) |
| 7 | 1.27 (0.89-1.81) | 1.21 (0.80-1.84) |
| 8 | 1.68 (1.19-2.37) | 1.70 (1.14-2.52) |
| P for trend | 0.642 | 0.699 |
| **Body fat index (kg/m^2^)** |  |  |
| Per SD increase | 1.09 (0.99 -1.19) | 1.11 (1.00-1.23) |
| Octiles^a^ |  |  |
| 1 | 1.00 | 1.00 |
| 2 | 1.39 (0.98-1.96) | 1.38 (0.93-2.05) |
| 3 | 1.23 (0.86-1.75) | 1.30 (0.87-1.95) |
| 4 | 1.53 (1.09-2.16) | 1.53 (1.03-2.27) |
| 5 | 158 (1.12-2.23) | 1.83 (1.25-2.70) |
| 6 | 1.56 (1.10-2.21) | 1.67 (1.12-2.49) |
| 7 | 1.53 (1.07-2.19) | 1.68 (1.12-2.54) |
| 8 | 1.69 (1.16-2.44) | 1.66 (1.08-2.56) |
| P for trend | 0.011 | 0.018 |
| **Trunk fat mass index (kg/m^2^)** |  |  |
| Per SD increase | 1.10 (1.01-1.20) | 1.11 (1.00-1.23) |
| Octiles^a^ |  |  |
| 1 | 1.00 | 1.00 |
| 2 | 1.67 (1.19-2.35) | 1.72 (1.16-2.53) |
| 3 | 1.30 (0.90-1.87) | 1.36 (0.89-2.06) |
| 4 | 1.42 (0.99-2.03) | 1.61 (1.07-2.42) |
| 5 | 1.66 (1.17-2.35) | 1.80 (1.20-2.68) |
| 6 | 1.66 (1.16-2.36) | 1.81 (1.20-2.72) |
| 7 | 1.86 (1.30-2.66) | 2.06 (1.37-3.11) |
| 8 | 1.70 (1.16-2.47) | 1.81 (1.17-2.81) |
| P for trend | 0.011 | 0.007 |

Adjusted for age at enrollment, education, race, family history of breast cancer, age at menarche, age at first full-term birth, parity, age at menopause, oral contraceptive use, hormone therapy use, physical activity, alcohol intake, smoking, lean mass and study component

^a^Cut-points- whole body fat mass (kg):- ≤20.39, 27.40-24.32, 24.33-27.72, 27.73-30.90, 30.91-34.53, 34.54-38.90,38.91-45.93, >45.93; whole body percent fat (kg): ≤35.5, 35.6-39.3, 39.4-42.0, 42.1-44.4, 44.5-46.6, 46.7-49.0, 49.1-52.0, >52, trunk fat mass (kg): ≤8.23, 8.24-10.65, 10.66-12.62, 12.63-14.43, 14.44-16.44, 16.45-18.85, 18.86-22.33,>22.33; fat mass of right leg (kg): <3.98, 3.99-4.66, 4.67-5.23, 5.24-5.81, 5.82-6.46, 6.47-7.24, 7.25-8.57, >8.57; fat mass of left leg (kg): <3.87, 3.88-4.54, 4.55-5.09, 5.10-5.67, 5.68-6.31, 6.32-7.08, 7.08-8.39, >8.39, ratio of trunk fat mass to average of R and L leg fat mass: ≤1.66, 1.66-1.98, 1.99-2.21, 2.22-2.43, 2.43-2.68, 2.68-2.96,2.97-3.39, >3.39; body fat index:- ≤7.85, 7.85-9.34, 9.35-10.64, 10.65-11.87, 11.88-13.29, 13.30-14.91, 14.92-17.54, >17.54; trunk fat mass index: ≤3.14, 3.15-4.09, 4.10-4.85, 4.86- 5.57, 5.58-6.31, 6.32-7.20, 7.20-8.58, >8.58 for octiles 1, 2, 3, 4, 5, 6, 7 and 8, respectively

**Table S5: Hazard ratios and 95% CI for the associations of baseline whole body fat mass to lean body mass ratio and incident, invasive breast cancer in postmenopausal women**

|  | Cases/person-year | HR (95% CI) |
| --- | --- | --- |
| **Overall** |  |  |
| Body fat to lean body mass ratio |  |  |
| Per SD increase |  | 1.13 (1.04-1.23) |
| Octiles^a^ |  |  |
| 1 | 60/19886.4 | 1.00 |
| 2 | 68/19986.4 | 1.16 (0.82-1.65) |
| 3 | 82/19820.3 | 1.46 (1.04-2.04) |
| 4 | 83/19837.8 | 1.51 (1.08-2.12) |
| 5 | 90/19934.9 | 1.63 (1.16-2.27) |
| 6 | 82/19156.7 | 1.54 (1.09-2.16) |
| 7 | 93/19398.1 | 1.74 (1.24-2.44) |
| 8 | 81/18442.6 | 1.61 (1.14-2.29) |
| *P for trend* |  | <0.001 |
| **ER+** |  |  |
| Body fat to lean body mass ratio^a^ |  |  |
| Per SD increase |  | 1.15 (1.05-1.27) |
| Octiles^a^ |  |  |
| 1 | 45/19886.4 | 1.00 |
| 2 | 52/19986.4 | 1.21 (0.82-1.81) |
| 3 | 65/19820.3 | 1.59 (1.09-2.34) |
| 4 | 61/19837.8 | 1.52 (1.03-2.26) |
| 5 | 70/19934.9 | 1.78 (1.21-2.61) |
| 6 | 63/19156.7 | 1.66 (1.12-2.45) |
| 7 | 64/19398.1 | 1.69 (1.14-2.51) |
| 8 | 64/18442.6 | 1.84 (1.24-2.75) |
| *P for trend* |  | <0.001 |

AdjAdjAdjusted for age at enrollment, education, race, family history of breast cancer, age at menarche, age at first full-term birth, parity, age at menopause, oral contraceptive use, physical activity, alcohol intake, smoking and study component

^a^Cutpoints- ≤0.579, 0.580-0.683, 0.684-0.763, 0.764-0.842, 0.843-0.919, 0.920-1.01, 1.02-1.15, >1.15

**Table S6: Hazard ratios and 95% CI for the associations of baseline whole body fat mass to lean body mass ratio and incident, invasive breast cancer in postmenopausal women from the Observational Study group**

|  | HR (95% CI) | HR (95% CI) |
| --- | --- | --- |
| **Overall** |  |  |
| Body fat to lean body mass ratio | 1.12 (1.00-1.25) | 1.12 (1.00-1.25) |
| Per SD increase |  |  |
| Octiles^a^ |  |  |
| 1 | 1.00 | 1.00 |
| 2 | 1.16 (0.82-1.65) | 1.16 (0.82-1.65) |
| 3 | 1.46 (1.04-2.04) | 1.46 (1.04-2.04) |
| 4 | 1.51 (1.08-2.12) | 1.51 (1.08-2.12) |
| 5 | 1.63 (1.16-2.27) | 1.63 (1.16-2.27) |
| 6 | 1.54 (1.09-2.16) | 1.54 (1.09-2.16) |
| 7 | 1.74 (1.24-2.44) | 1.74 (1.24-2.44) |
| 8 | 1.61 (1.14-2.29) | 1.61 (1.14-2.29) |
| *P for trend* | <0.001 | <0.001 |
| **ER+** |  |  |
| Body fat to lean body mass ratio |  |  |
| Per SD increase | 1.16 (1.03-1.33) | 1.16 (1.03-1.33) |
| Octiles^a^ |  |  |
| 1 | 1.00 | 1.00 |
| 2 | 0.99 (0.60-1.63) | 0.99 (0.60-1.63) |
| 3 | 1.54 (0.98-2.44) | 1.54 (0.98-2.44) |
| 4 | 1.39 (0.86-2.24) | 1.39 (0.86-2.24) |
| 5 | 1.72 (1.08-2.74) | 1.72 (1.08-2.74) |
| 6 | 1.40 (0.85-2.31) | 1.40 (0.85-2.31) |
| 7 | 1.64 (1.00-2.67) | 1.64 (1.00-2.67) |
| 8 | 1.84 (1.24-2.75) | 1.84 (1.24-2.75) |
| *P for trend* | <0.001 | <0.001 |

Adjusted for age at enrollment, education, race, family history of breast cancer, age at menarche, age at first full-term birth, parity, age at menopause, oral contraceptive use, physical activity, alcohol intake, smoking and study component

^a^Cutpoints- ≤0.579, 0.580-0.683, 0.684-0.763, 0.764-0.842, 0.843-0.919, 0.920-1.01, 1.02-1.15, >1.15

**Table S7: Hazard ratios for the association between baseline body fat measures and incident, invasive breast cancer by hormone therapy (HT) use in postmenopausal women^a^**

|  | HT use | | | | | |
| --- | --- | --- | --- | --- | --- | --- |
|  | Never | | Former | | Current | |
|  | No. of cases | HR (95% CI) | No. of cases | HR (95% CI) | No. of cases | HR (95% CI) |
| Whole body fat mass (kg)^b^ |  |  |  |  |  |  |
| Per SD increase |  | 1.24 (1.11-1.38) |  | 1.11 (0.88-1.41) |  | 1.20 (1.05-1.38) |
| 1 | 34 | 1.00 | 13 | 1.00 | 51 | 1.00 |
| 2 | 54 | 1.46 (0.94-2.27) | 15 | 0.89 (0.42-1.92) | 58 | 1.02 (0.68-1.53) |
| 3 | 47 | 1.54 (1.00-2.37) | 22 | 1.56 (0.80-3.06) | 52 | 1.57 (1.07-2.30) |
| 4 | 79 | 1.92 (1.27-2.91) | 13 | 0.68 (0.29-1.56) | 44 | 1.42 (0.94-2.15) |
| 5 | 88 | 2.20 (1.46-3.31) | 18 | 1.45 (0.69-3.02) | 50 | 1.46 (0.95-2.26) |
| *P for trend* |  | <0.001 |  | 0.346 |  | 0.035 |
| *P for heterogeneity* |  | 0.270 | | |  |  |
|  |  |  |  |  |  |  |
| Whole body fat percent^b^ |  |  |  |  |  |  |
| Per SD increase |  | 1.21 (1.07-1.37) |  | 1.07 (0.84-1.35) |  | 1.14 (0.99-1.31) |
| 1 | 37 | 1.00 | 16 | 1.00 | 56 | 1.00 |
| 2 | 55 | 1.40 (0.92-2.13) | 14 | 0.96 (0.46-1.98) | 48 | 0.97 (0.65-1.44) |
| 3 | 67 | 1.80 (1.19-2.70) | 18 | 1.13 (0.57-2.26) | 57 | 1.27 (0.87-1.85) |
| 4 | 70 | 1.73 (1.15-2.60) | 14 | 0.84 (0.40-1.76) | 50 | 1.25 (0.84-1.86) |
| 5 | 73 | 1.77 (1.17-2.68) | 19 | 1.31 (0.65-2.65) | 44 | 1.33 (0.88-2.03) |
| *P for trend* |  | 0.005 |  | 0.591 |  | 0.094 |
| *P for heterogeneity* |  | 0.576 | | |  |  |
|  |  |  |  |  |  |  |
| Trunk fat mass (kg)^b^ |  |  |  |  |  |  |
| Per SD increase |  | 1.24 (1.11-1.39) |  | 1.05 (0.83-1.32) |  | 1.23 (1.07-1.40) |
| 1 | 14 | 1.00 | 37 | 1.00 | 37 | 1.00 |
| 2 | 30 | 1.66 (1.08-2.57) | 48 | 1.05 (0.49-2.24) | 48 | 1.25 (0.85-1.83) |
| 3 | 37 | 1.30 (0.83-2.03) | 43 | 1.56 (0.78-3.16) | 43 | 1.33 (0.89-1.97) |
| 4 | 19 | 2.14 (1.41-3.23) | 43 | 0.93 (0.42-2.05) | 43 | 1.29 (0.85-1.96) |
| 5 | 44 | 2.15 (1.42-3.25) | 51 | 1.48 (0.70-3.15) | 51 | 1.90 (1.25-2.89) |
| *P for trend* |  | <0.001 |  | 0.404 |  | 0.004 |
| *P for heterogeneity* |  | 0.604 | | |  |  |
|  |  |  |  |  |  |  |
| Fat mass of right leg (kg)^b^ |  |  |  |  |  |  |
| Per SD increase |  | 1.22 (1.09-1.35) |  | 1.11 (0.88-1.40) |  | 1.11 (0.97-1.27) |
| 1 | 44 | 1.00 | 13 | 1.00 | 43 | 1.00 |
| 2 | 39 | 0.85 (0.55-1.32) | 15 | 1.37 (0.64-2.92) | 56 | 1.21 (0.81-1.81) |
| 3 | 61 | 1.39 (0.94-2.06) | 19 | 1.50 (0.73-3.10) | 59 | 1.21 (0.81-1.80) |
| 4 | 67 | 1.41 (0.96-2.08) | 16 | 1.31 (0.62-2.76) | 47 | 1.16 (0.76-1.77) |
| 5 | 91 | 1.83 (1.26-2.65) | 18 | 1.60 (0.75-3.39) | 50 | 1.37 (0.90-2.10) |
| *P for trend* |  | <0.001 |  | 0.313 |  | 0.219 |
| *P for heterogeneity* |  | 0.031 | | |  |  |
|  |  |  |  |  |  |  |
| Fat mass of left leg (kg)^b^ |  |  |  |  |  |  |
| Per SD increase |  | 1.21 (1.08-1.34) |  | 1.08 (0.86-1.37) |  | 1.12 (0.98-1.28) |
| 1 | 44 | 1.00 | 13 | 1.00 | 43 | 1.00 |
| 2 | 39 | 0.83 (0.54-1.27) | 15 | 1.36 (0.64-2.89) | 56 | 1.30 (0.87-1.95) |
| 3 | 61 | 1.27 (0.86-1.88) | 19 | 1.59 (0.78-3.26) | 59 | 1.26 (0.84-1.89) |
| 4 | 67 | 1.30 (0.89-1.91) | 16 | 1.20 (0.56-2.57) | 47 | 1.20 (0.78-1.85) |
| 5 | 91 | 1.77 (1.22-2.55) | 18 | 1.62 (0.76-3.43) | 50 | 1.53 (0.99-2.34) |
| *P for trend* |  | <0.001 |  | 0.336 |  | 0.105 |
| *P for heterogeneity* |  |  |  | 0.105 |  |  |
|  |  |  |  |  |  |  |
| Ratio of trunk fat mass to average of R and L leg fat mass^b^ |  |  |  |  |  |  |
| Per SD increase |  | 1.10 (0.98-1.72) |  | 0.93 (0.73-1.19) |  | 1.14 (1.00-1.30) |
| 1 | 32 | 1.00 | 18 | 1.00 | 50 | 1.00 |
| 2 | 74 | 2.12 (1.40-3.21) | 13 | 0.65 (0.32-1.34) | 58 | 1.35 (0.92-1.97) |
| 3 | 65 | 1.85 (1.20-2.83) | 18 | 0.84 (0.43-1.64) | 52 | 1.33 (0.90-1.97) |
| 4 | 59 | 1.65 (1.07-2.55) | 18 | 0.93 (0.47-1.83) | 52 | 1.52 (1.02-2.25) |
| 5 | 72 | 1.95 (1.27-2.98) | 14 | 0.74 (0.36-1.52) | 43 | 1.58 (1.04-2.40) |
| *P for trend* |  | 0.054 |  | 0.679 |  | 0.027 |
| *P for heterogeneity* |  | 0.598 | | |  |  |
|  |  |  |  |  |  |  |
| Body fat index (kg/m^2^)^b^ |  |  |  |  |  |  |
| Per SD increase |  | 1.20 (1.08-1.34) |  | 1.10 (0.86-1.39) |  | 1.16 (1.02-1.32) |
| 1 | 34 | 1.00 | 17 | 1.00 | 51 | 1.00 |
| 2 | 43 | 1.30 (0.82-2.04) | 12 | 0.78 (0.37-1.65) | 55 | 1.16 (0.79-1.70) |
| 3 | 59 | 1.70 (1.11-2.62) | 22 | 1.14 (0.59-2.19) | 51 | 1.25 (0.84-1.87) |
| 4 | 74 | 1.93 (1.27-2.94) | 11 | 0.68 (0.31-1.50) | 58 | 1.66 (1.12-2.47) |
| 5 | 90 | 2.24 (1.48-3.39) | 19 | 1.29 (0.64-2.60) | 40 | 1.46 (0.94-2.28) |
| *P for trend* |  | <0.001 |  | 0.492 |  | 0.025 |
| *P for heterogeneity* |  | 0.246 | | |  |  |
|  |  |  |  |  |  |  |
| Trunk fat mass index (kg/m^2^)^b^ |  |  |  |  |  |  |
| Per SD increase |  | 1.21 (1.09-1.35) |  | 1.05 (0.83-1.34) |  | 1.19 (1.05-1.36) |
| 1 | 15 | 1.00 | 39 | 1.00 |  | 1.00 |
| 2 | 35 | 1.74 (1.10-2.73) | 54 | 0.87 (0.42-1.80) |  | 1.26 (0.87-1.85) |
| 3 | 25 | 1.65 (1.05-2.59) | 41 | 1.29 (0.67-2.49) |  | 1.23 (0.83-1.84) |
| 4 | 28 | 2.50 (1.64-3.83) | 44 | 0.49 (0.20-1.16) |  | 1.38 (0.91-2.08) |
| 5 | 37 | 2.32 (1.50-3.57) | 47 | 1.44 (0.71-2.90) |  | 1.78 (1.16-2.72) |
| *P for trend* |  | <0.001 |  | 0.488 |  | 0.010 |
| *P for heterogeneity* |  | 0.397 | | |  |  |

^a^Adjusted for age at enrollment, education, race/ethnicity, family history of breast cancer, age at menarche, age at first full-term birth, parity, age at menopause, oral contraceptive use, physical activity, alcohol intake, and smoking

^b^Cut-points- whole body fat mass (kg):- ≤22.87, 22.88-28.35, 28.36-33.82, 33.83-41.38, >41.38; whole body percent fat (kg): ≤38.1, 38.2-42.5, 42.6-46.1, 46.2-50.1, >50.1, trunk fat mass (kg): ≤9.74, 9.75-12.98, 12.99-16.01, 16.02-20.01, >20.01; fat mass of right leg (kg): <4.41, 4.42-5.34, 5.35-6.33, 6.34-7.69, >7.69; fat mass of left leg (kg): <4.31, 4.32-5.21, 5.22-6.16, 6.17-7.53, >7.53; ratio of trunk fat mass to average of R and L leg fat mass: ≤1.87, 1.88-2.25, 2.26-2.63, 2.64-3.12, >3.12; body fat index:- ≤8.80, 8.81-10.89, 10.90-12.97, 12.98-15.79, >15.79; trunk fat mass index: ≤3.73, 3.74-5.00, 5.01-6.16, 6.17-7.66, >7.66 for quintiles 1, 2, 3, 4 and 5, respectively

**Table S8: Hazard ratios and 95% CI from time-dependent analyses for the association between anthropometric measures and incident breast cancer in postmenopausal women**

|  | Overall | ER-positive |
| --- | --- | --- |
|  | HR (95% CI) | |
| BMI (kg/m^2^) |  |  |
| Octiles^a^ |  |  |
| 1 | 1.00 | 1.00 |
| 2 | 0.99 (0.71-1.39) | 1.01 (0.69-1.48) |
| 3 | 1.15 (0.82-1.59) | 1.11 (0.76-1.62) |
| 4 | 1.15 (0.82-1.61) | 1.06 (0.72-1.56) |
| 5 | 1.26 (0.91-1.75) | 1.25 (0.86-1.83) |
| 6 | 1.42 (1.03-1.97) | 1.49 (1.03-2.15) |
| 7 | 1.52 (1.09-2.10) | 1.77 (1.23-2.54) |
| 8 | 1.70 (1.23-2.35) | 1.66 (1.14-2.42) |
| *P for trend* | <0.001 | <0.001 |
|  |  |  |
| Waist circumference (cm.) |  |  |
| Octiles^a^ |  |  |
| 1 | 1.00 | 1.00 |
| 2 | 1.07 (0.75-1.53) | 0.97 (0.65-1.44) |
| 3 | 1.17 (0.83-1.65) | 1.19 (0.82-1.74) |
| 4 | 1.17 (0.82-1.67) | 0.98 (0.65-1.48) |
| 5 | 1.37 (0.97-1.93) | 1.08 (0.72-1.61) |
| 6 | 1.88 (1.34-2.62) | 2.07 (1.44-2.98) |
| 7 | 1.58 (1.12-2.23) | 1.55 (1.06-2.28) |
| 8 | 1.81 (1.29-2.54) | 1.58 (1.07-2.32) |
| *P for trend* | <0.001 | <0.001 |
|  |  |  |
| WHR |  |  |
| Octiles^a^ |  |  |
| 1 | 1.00 | 1.00 |
| 2 | 1.49 (1.05-2.12) | 1.25 (0.84-1.85) |
| 3 | 1.60 (1.13-2.27) | 1.49 (1.02-2.17) |
| 4 | 1.61 (1.13-2.27) | 1.43 (0.98-2.10) |
| 5 | 1.54 (1.08-2.19) | 1.27 (0.86-1.90) |
| 6 | 1.95 (1.38-2.74) | 1.69 (1.16-2.47) |
| 7 | 1.66 (1.16-2.36) | 1.45 (0.98-2.14) |
| 8 | 1.63 (1.14-2.32) | 1.46 (0.98-2.16) |
| *P for trend* | 0.008 | 0.051 |

Adjusted for age at enrollment, education, race/ethnicity, family history of breast cancer, personal history of diabetes, age at menarche, age at first full-term birth, parity, age at menopause, oral contraceptive use, hormone therapy, physical activity, alcohol intake, and study component

^a^Cutpoints- BMI (kg/m^2^): ≤22.15, 22.16-24.00, 24.01-25.60, 25.61-27.19, 27.20-29.07, 29.08-31.31, 31.32-34.87, >34.87; waist circumference (cm): ≤71, 72-76, 77-80, 81-84, 85-89, 90-94, 95-102, >102; waist to hip ratio: ≤0.723, 0.724-.751, 0.752-0.774, 0.775-0.797, 0.798-0.821, 0.822-0.849, 0.850-0.890, >0.890 for octiles 1, 2, 3, 4, 5, 6, 7 and 8, respectively

**Table S9: Hazard ratios for the association of baseline body fat and incident breast cancer excluding women with a breast cancer diagnosis within two years of enrollment**

|  | Breast cancer | |
| --- | --- | --- |
|  | Overall | ER+ |
|  | HR (95% CI) | |
| Whole body fat mass (kg) |  |  |
| Octiles^a^ |  |  |
| 1 | 1.00 | 1.00 |
| 2 | 1.09 (0.75-1.60) | 1.05 (0.68-1.61) |
| 3 | 1.45 (1.01-2.09) | 1.30 (0.86-1.97) |
| 4 | 1.29 (0.89-1.87) | 1.33 (0.88-2.01) |
| 5 | 1.85 (1.30-2.62) | 1.93 (1.31-2.85) |
| 6 | 1.48 (1.03-2.14) | 1.67 (1.11-2.49) |
| 7 | 1.84 (1.29-2.63) | 1.82 (1.21-2.72) |
| 8 | 2.08 (1.46-2.96) | 2.15 (1.44-3.21) |
| *P for trend* | <0.001 | <0.001 |
|  |  |  |
| Whole body fat percent |  |  |
| Octiles^a^ |  |  |
| 1 | 1.00 | 1.00 |
| 2 | 1.00 (0.69-1.46) | 1.03 (0.67-1.257) |
| 3 | 1.42 (1.00-2.00) | 1.50 (1.01-2.23) |
| 4 | 1.47 (1.04-2.08) | 1.52 (1.02-2.26) |
| 5 | 1.55 (1.10-2.20) | 1.79 (1.21-2.65) |
| 6 | 1.41 (0.98-2.02) | 1.56 (1.04-2.34) |
| 7 | 1.80 (1.28-2.53) | 1.80 (1.21-2.67) |
| 8 | 1.54 (1.07-2.20) | 1.89 (1.26-2.82) |
| *P for trend* | <0.001 | <0.001 |
|  |  |  |
| Trunk fat mass (kg) |  |  |
| Octiles^a^ |  |  |
| 1 | 1.00 | 1.00 |
| 2 | 1.50 (1.03-2.20) | 1.57 (1.02-2.41) |
| 3 | 1.83 (1.27-2.66) | 1.82 (1.20-2.78) |
| 4 | 1.32 (0.89-1.97) | 1.29 (0.82-2.04) |
| 5 | 2.21 (1.54-3.18) | 2.42 (1.62-3.64) |
| 6 | 2.05 (1.41-2.98) | 2.21 (1.45-3.37) |
| 7 | 1.82 (1.24-2.67) | 2.08 (1.35-3.19) |
| 8 | 2.66 (1.84-3.82) | 2.68 (1.76-4.06) |
| *P for trend* | <0.001 | <0.001 |
|  |  |  |
| Fat mass of right leg (kg) |  |  |
| Octiles^a^ |  |  |
| 1 | 1.00 | 1.00 |
| 2 | 1.14 (0.79-1.65) | 1.07 (0.70-1.64) |
| 3 | 1.14 (0.79-1.64) | 1.07 (0.70-1.64) |
| 4 | 1.29 (0.90-1.85) | 1.35 (0.90-2.02) |
| 5 | 1.29 (0.90-1.85) | 1.43 (0.96-2.14) |
| 6 | 1.40 (0.98-2.00) | 1.52 (1.02-2.28) |
| 7 | 1.43 (1.00-2.04) | 1.64 (1.11-2.44) |
| 8 | 1.89 (1.34-2.68) | 1.89 (1.27-2.81) |
| *P for trend* | <0.001 | <0.001 |
|  |  |  |
| Fat mass of left leg (kg) |  |  |
| Octiles^a^ |  |  |
| 1 | 1.00 | 1.00 |
| 2 | 1.12 (0.77-1.61) | 1.01 (0.66-1.54) |
| 3 | 1.18 (0.82-1.70) | 1.12 (0.74-1.69) |
| 4 | 1.24 (0.86-1.77) | 1.22 (0.81-1.83) |
| 5 | 1.38 (0.97-1.96) | 1.48 (1.00-2.19) |
| 6 | 1.37 (0.96-1.95) | 1.49 (1.01-2.21) |
| 7 | 1.50 (1.06-2.13) | 1.57 (1.06-2.33) |
| 8 | 1.72 (1.21-2.43) | 1.67 (1.12-2.49) |
| *P for trend* | <0.001 | <0.001 |
|  |  |  |
| Ratio of trunk fat mass to average of R and L leg fat mass |  |  |
| Octiles^a^ |  |  |
| 1 | 1.00 | 1.00 |
| 2 | 1.34 (0.94-1.92) | 1.27 (0.84-1.92) |
| 3 | 1.62 (1.15-2.29) | 1.87 (1.28-2.75) |
| 4 | 1.53 (1.07-2.18) | 1.58 (1.06-2.37) |
| 5 | 1.45 (1.01-2.08) | 1.39 (0.91-2.10) |
| 6 | 1.59 (1.11-2.28) | 1.74 (1.16-2.60) |
| 7 | 1.48 (1.02-2.13) | 1.46 (0.96-2.22) |
| 8 | 1.92 (1.35-2.74) | 1.98 (1.32-2.97) |
| *P for trend* | 0.003 | 0.009 |
|  |  |  |
| Body fat index |  |  |
| Octiles^a^ |  |  |
| 1 | 1.00 | 1.00 |
| 2 | 1.33 (0.92-1.92) | 1.34 (0.88-2.04) |
| 3 | 1.22 (0.83-1.78) | 1.30 (0.84-1.99) |
| 4 | 1.61 (1.12-2.30) | 1.60 (1.06-2.41) |
| 5 | 1.74 (1.22-2.49) | 2.06 (1.38-3.06) |
| 6 | 1.61 (1.12-2.32) | 1.82 (1.21-2.75) |
| 7 | 1.78 (1.23-2.55) | 2.00 (1.33-3.02) |
| 8 | 2.11 (1.47-3.01) | 2.17 (1.44-3.28) |
| *P for trend* | <0.001 | <0.001 |
|  |  |  |
| Trunk fat mass index |  |  |
| Octiles^a^ |  |  |
| 1 | 1.00 | 1.00 |
| 2 | 1.56 (1.09-2.24) | 1.66 (1.10-2.51) |
| 3 | 1.36 (0.93-2.00) | 1.42 (0.92-2.21) |
| 4 | 1.47 (1.01-2.15) | 1.69 (1.10-2.59) |
| 5 | 1.80 (1.25-2.60) | 1.96 (1.29-2.98) |
| 6 | 1.90 (1.31-2.74) | 2.09 (1.37-3.18) |
| 7 | 2.09 (1.45-3.01) | 2.40 (1.59-3.64) |
| 8 | 2.22 (1.54-3.20) | 2.46 (1.61-3.75) |
| *P for trend* | <0.001 | <0.001 |

Adjusted for age at enrollment, education, race/ethnicity, family history of breast cancer, age at menarche, age at first full-term birth, parity, age at menopause, oral contraceptive use, hormone therapy use, physical activity, alcohol intake, smoking and study component

^a^Cut-points- whole body fat mass (kg):- ≤20.39, 27.40-24.32, 24.33-27.72, 27.73-30.90, 30.91-34.53, 34.54-38.90,38.91-45.93, >45.93; whole body percent fat (kg): ≤35.5, 35.6-39.3, 39.4-42.0, 42.1-44.4, 44.5-46.6, 46.7-49.0, 49.1-52.0, >52, trunk fat mass (kg): ≤8.23, 8.24-10.65, 10.66-12.62, 12.63-14.43, 14.44-16.44, 16.45-18.85, 18.86-22.33,>22.33; fat mass of right leg (kg): <3.98, 3.99-4.66, 4.67-5.23, 5.24-5.81, 5.82-6.46, 6.47-7.24, 7.25-8.57, >8.57; fat mass of left leg (kg): <3.87, 3.88-4.54, 4.55-5.09, 5.10-5.67, 5.68-6.31, 6.32-7.08, 7.08-8.39, >8.39 for octiles 1, 2, 3, 4, 5, 6, 7 and 8, respectively
